# A systematic review of behaviour change techniques within interventions to prevent return to smoking postpartum

**DOI:** 10.1016/j.addbeh.2018.12.031

**Published:** 2019-05

**Authors:** Tracey J. Brown, Wendy Hardeman, Linda Bauld, Richard Holland, Vivienne Maskrey, Felix Naughton, Sophie Orton, Michael Ussher, Caitlin Notley

**Affiliations:** a Norwich Medical School, University of East Anglia, Norwich, UK; b School of Health Sciences, University of East Anglia, Norwich, UK; c Usher Institute, College of Medicine and Veterinary Medicine, University of Edinburgh, Edinburgh, UK; d Leicester Medical School, University of Leicester, Leicester, UK; e Division of Primary Care, University of Nottingham, Nottingham, UK; f Population Health Research Institute, St George's, University of London, London, UK & Institute for Social Marketing, University of Stirling, Stirling, UK

**Keywords:** Smoking relapse, Intervention, Postpartum, Pregnancy, Review, Behaviour change techniques

## Abstract

**Introduction:**

There is no routine support to prevent postpartum smoking relapse, due to lack of effective interventions. Previous reviews have identified behaviour change techniques (BCTs) within pregnancy cessation trials to specify which components might be incorporated into more effective interventions, but no reviews have identified BCTs for prevention of smoking relapse postpartum. We reviewed BCTs and potential delivery modes, to inform future interventions.

**Methods:**

We searched Medline and EMBASE from January 2015–May 2017; and identified trials published before 2015 by handsearching systematic reviews. We included RCTs where: i) ≥1 intervention component aimed to maintain smoking abstinence versus a less intensive intervention; ii) participants included pregnant or postpartum smoking quitters; iii) smoking status was reported in the postpartum period. We extracted trial characteristics and used the Behaviour Change Technique Taxonomy v1 to extract BCTs. We aimed to identify ‘promising’ BCTs i.e. those frequently occurring and present in ≥2 trials that demonstrated long-term effectiveness (≥6 months postpartum). Data synthesis was narrative.

**Results:**

We included 32 trials, six of which demonstrated long-term effectiveness. These six trials used self-help, mainly in conjunction with counselling, and were largely delivered remotely. We identified six BCTs as promising: ‘problem solving’, ‘information about health consequences’, ‘information about social and environmental consequences’, ‘social support’, ‘reduce negative emotions’ and ‘instruction on how to perform a behaviour’.

**Conclusions:**

Future interventions to prevent postpartum smoking relapse might include these six BCTs to maximise effectiveness. Tailored self-help approaches, with/without counselling, may be favourable modes of delivery of BCTs.

Registration: PROSPERO CRD42018075677.

## Highlights

• First review of behaviour change techniques to prevent postpartum smoking relapse.
• Six promising behaviour change techniques (BCTs) were frequently coded.
• BCTs were problem solving, social support, information about consequences.
• How to perform a behaviour and reduce negative emotions were also promising BCTs.

## 1 Introduction

Smoking in pregnancy or postpartum remains a major preventable cause of maternal and infant mortality and morbidity (DoH, 2017). Pregnancy acts as a strong motivator for smoking cessation, with more women quitting during pregnancy than at any other time in the life course. Indeed, it is estimated that up to 49% of women are able to ‘spontaneously quit’ before their first antenatal appointment (Chamberlain, O'Mara-Eves, Porter, et al., 2017) due to factors such as maternal concerns for the health of the unborn baby (Flemming, McCaughan, Angus, & Graham, 2015; Notley, Blyth, Craig, Edwards, & Holland, 2015). Pregnant women can be considered a particularly vulnerable population, since not only are they themselves at risk of harm from tobacco smoke, but there are also increased health risks to the developing fetus and to infants born to smoking mothers. Cessation support, including the UK NHS Smokefree services and provision of carbon monoxide monitoring, are effective and cost effective ways to support pregnant smokers to quit (NICE, 2010). Most women who quit smoking during pregnancy wish to remain abstinent, but a high proportion return to smoking postpartum (Chamberlain et al., 2017; Jones, Lewis, Parrott, Wormall, & Coleman, 2016; Lumley et al., 2009). Postpartum women are particularly vulnerable to smoking relapse due to factors such as perception of no longer needing to protect the baby, stress of dealing with a new infant, lack of confidence in remaining quit, nicotine dependence, living with a smoking partner, and a desire to return to their pre-pregnancy identity (Flemming et al., 2015; Notley et al., 2015; Orton, Coleman, Coleman-Haynes, & Ussher, 2018; Riaz, Lewis, Naughton, & Ussher, 2018). Rates of return to smoking in the literature vary due to specific population characteristics and variation in success of any provided intervention (Fang, Goldstein, Butzen, et al., 2004; Jones et al., 2016). Recent data estimates that up to 76% of spontaneous quitters restart smoking postpartum (Jones et al., 2016). For women receiving cessation support; who may be more addicted or have weaker beliefs about the dangers of smoking; rates may be higher (Chamberlain et al., 2017; Jones et al., 2016; Lumley et al., 2009). In randomised clinical trials of within–pregnancy smoking cessation interventions, secondary analysis using point-prevalence data estimated that across trials, the mean proportion of women smoking at the end of pregnancy was 87%, rising to 94% six months later, suggesting the majority of smoking cessation trial participants continue to smoke both throughout pregnancy and after childbirth (Jones et al., 2016).

Return to smoking postpartum has substantial health and cost implications, thus reducing exposure to smoke in pregnancy is an important goal worldwide (WHO, 2013). In the UK alone, the annual cost to the NHS of continuing to smoke in pregnancy is estimated to be as high as £64 million for treating health related problems for mothers; and costs for treating mortality and morbidity of infants (aged 0–12 months) due to maternal smoking an estimated further £23.5 million (Godfrey, Pickett, Parrott, Mdege, & Eapen, 2010). Sustained smoking abstinence postpartum has significant health benefits for the mother and wider family due to reduced exposure to second hand smoke. Quitting smoking before the age of 30 avoids >97% of the excess mortality caused by smoking, due to lower risks of cancers, coronary heart disease and cerebrovascular disease (Pirie, Peto, Reeves, Green, & Beral, 2013). Infants exposed to second hand smoke have a higher incidence of sudden infant death, respiratory conditions including asthma, bronchitis and pneumonia, and other infections such as middle ear disease and meningitis (RCP, 2010). Furthermore, children of smoking mothers are twice as likely to become smokers themselves, perpetuating the cycle (Leonardi-Bee, Jere, & Britton, 2011). The prevalence of smoking during pregnancy is higher in more disadvantaged groups (Chamberlain et al., 2017; Coleman, Chamberlain, Davey, Cooper, & Leonardi-Bee, 2015) and being less well educated has been identified as an important predictor of relapse postpartum (Orton et al., 2018). Despite these inequalities, health and cost implications; there is no routine provision of support to prevent relapse due to a lack of evidence-based interventions (Hajek et al., 2013). There is thus a clear need to develop an effective intervention to prevent return to smoking in postpartum women.

A number of interventions for relapse prevention in pregnancy and maintenance into the postpartum period have been developed; with modest success for those incorporating counselling, health education and/or financial incentives (Chamberlain et al., 2017). Behavioural change interventions are complex in nature, consisting of multiple components designed to affect change (Craig et al., 2008). In order to describe active ingredients (components) of interventions, Michie and colleagues developed the Behaviour Change Technique Taxonomy v1 (BCTTv1) (Michie et al., 2015; Michie, Richardson, Johnston, et al., 2013). They propose that by identifying individual behaviour change techniques (BCTs) within interventions, it is possible to specify how interventions operate and therefore what components might be incorporated into new and more effective interventions (Michie et al., 2015). Other reviews have identified BCTs for a range of behaviour change interventions including smoking cessation support for general smokers (Lorencatto, West, Seymour, & Michie, 2013; West, Walia, Hyder, Shahab, & Michie, 2010) and for smoking cessation in pregnancy (Campbell, Fergie, Coleman-Haynes, et al., 2018; Lorencatto, West, & Michie, 2012); but no reviews have identified BCTs for smoking relapse prevention in the postpartum period. We used the BCTTv1 taxonomy (Michie et al., 2013) to identify BCT components which could be considered ‘promising, or likely to be effective in future interventions to maintain smoking abstinence postpartum. We also reviewed the characteristics (mode of delivery and fidelity) of smoking relapse prevention interventions in order to understand how these ‘promising’ BCTs might be best delivered.

## 2 Methods

### 2.1 Types of trials

We included randomised controlled trials of interventions where at least one component was designed to maintain smoking abstinence, versus a less intensive intervention or usual care. Participants were generally healthy pregnant or postpartum women who were recent smoking quitters (or a mixed population of recent quitters and smokers at baseline). Trials had to report smoking status (self-report or biochemically validated) in the postpartum period (beyond delivery). Trials not published in English were excluded due to the detailed intervention information required. Our review was registered on the PROSPERO international prospective register of systematic reviews (CRD42018075677).

### 2.2 Searches

We searched Medline and EMBASE from 1st January 2015 to 9th May 2017, to replicate and update the search strategy used by Jones and colleagues (Jones et al., 2016) in a published systematic review of restarting smoking in the postpartum period. Keywords included: pregnancy, antenatal, prenatal, childbirth, postnatal, postpartum, breastfeeding, fetus, newborn, infant, tobacco, smoking, smoking cessation, and relapse. The full search strategy is published in Jones. (Jones et al., 2016)

Randomised controlled trials published prior to 2015 were identified by handsearching Jones (Jones et al., 2016) and other recent pertinent systematic reviews of smoking cessation in pregnancy (Chamberlain et al., 2017; Coleman et al., 2015) and postpartum relapse prevention interventions (Notley et al., 2015; Orton et al., 2018).

### 2.3 Selection of trials

One author (TJB) screened citations on the basis of title and abstract, and also using tables of study characteristics when handsearching. A second reviewer (IG) independently screened 10% of titles and abstracts for studies that appeared to be relevant to the review. Disagreement rate was not formally measured, but any disagreements were resolved by consensus. Where it was unclear if a study met our inclusion criteria, the full-text was collected and assessed. Each full-text article was assessed for inclusion using an inclusion/exclusion log. For excluded studies, the reason for exclusion was recorded at full-text assessment.

### 2.4 Data extraction

We identified BCTs targeting smoking behaviour, i.e. BCTs included to maintain smoking abstinence, or to promote smoking cessation together with maintaining smoking abstinence. The BCTTv1 was used to extract BCTs by assigning BCT codes to appropriate sections of trial articles (Michie et al., 2013). Two researchers (TJB and CN) completed online training in BCTTv1 use (www.bct-taxonomy.com). To ensure consistency in data recording, data extraction of BCTs was duplicated independently at a level of 10%, with any disagreements resolved by discussion or the involvement of an expert third reviewer (WH) who co-authored the BCTTv1 (Michie et al., 2013; Michie et al., 2015). To ensure any relevant BCTs were captured, we extracted BCTs whether they were definitely (coded ++) or probably (coded +) present, following BCTTv1 coding principles (www.bct-taxonomy.com). We calculated frequency of occurrence of BCTs across trials to define a list of ‘promising’ BCTs, most likely to be effective in relapse prevention interventions. We defined a BCT as ‘promising’ if it was present in at least two long-term effective interventions, as in Lorencatto (Lorencatto et al., 2012), *and* where frequency in all included trials, regardless of trial effectiveness, was highest (present in ≥25% interventions). We defined long-term effective interventions as those finding statistically significant (*p* < .05 where reported) differences in smoking abstinence between the intervention and control groups at six months postpartum or later (biochemically validated or self-report). The rationale for this two-pronged approach, was that by considering BCTs in light of their overall frequency, we might identify techniques most likely to be feasible, acceptable and fit for purpose.

We also extracted data concerning characteristics of trial participants, details of the intervention and control, smoking status outcomes and data on intervention fidelity (adherence to the protocol, acceptability, compliance) in order to consider BCTs which might be most effective and suitable for postpartum relapse interventions and how these interventions might be best delivered. Data synthesis was narrative.

## 3 Results

### 3.1 Results of the search

**Fig. 1:**
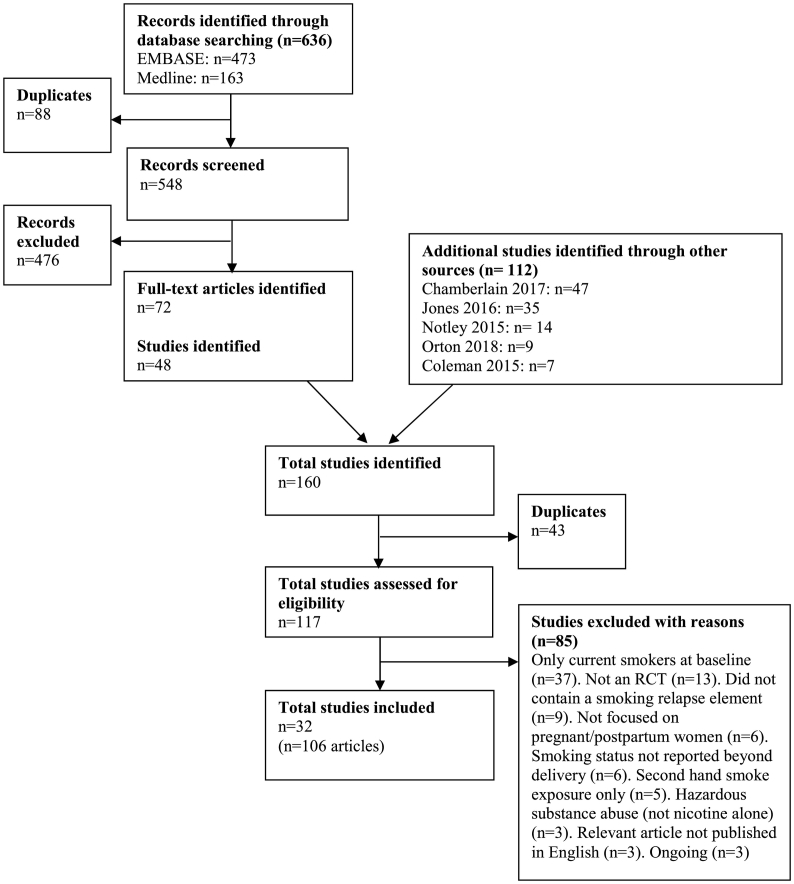
Flow diagram.

The inclusion of RCTs is summarised in Fig. 1. Electronic searching identified 636 records, 88 duplicate references were removed, resulting in 548 references for title and abstract screening. From these records, 72 relevant full text articles were identified (48 unique trials). Handsearching identified a further 69 relevant trials (after removal of duplicates). In total, articles from 117 studies were retrieved for full text assessment with the final inclusion of 32 primary RCTs (106 articles : Supplementary Table 1) which satisfied all inclusion criteria (Allen, Allen, Lunos, & Tosun, 2016; Brandon, Simmons, Meade, et al., 2012; Cummins, Tedeschi, Anderson, & Zhu, 2016; Edwards & Sims-Jones, 1997; El-Mohandes, El-Khorazaty, Kiely, & Gantz, 2011; Ershoff, Quinn, & Mullen, 1995; Forray, Gilstad-Hayden, Sofuoglu, & Yonkers, 2016; Forray & Waters, 2015; Hajek, West, Lee, et al., 2001; Hannover, Thyrian, Roske, et al., 2009; Jimenez-Muro, Nerin, Samper, et al., 2013; Johnson, Ratner, Bottorff, Hall, & Dahinten, 2000; Kendrick, Zahniser, Miller, et al., 1995; Kientz & Kupperschmidt, 2005;Levine, Cheng, Marcus, Kalarchian, & Emery, 2016; Lillington, Royce, Novak, Ruvalcaba, & Chlebowski, 1995; McBride et al., 1999; McBride, Baucom, Peterson, et al., 2004; Morasco, Dornelas, Fischer, Oncken, & Lando, 2006; Mullen, DiClemente, & Bartholomew, 2001; Pbert, Ockene, Zapka, et al., 2004; Petersen, Handel, Kotch, Podedworny, & Rosen, 1992; Polanska, Hanke, Sobala, & Lowe, 2004; Pollak, Fish, Lyna, et al., 2016; Reitzel, Vidrine, Businelle, et al., 2010; Ruger, Weinstein, Hammond, Kearney, & Emmons, 2008; Secker-Walker, Solomon, Flynn, et al., 1995; Strecher et al., 2000; Secker-Walker, Solomon, Flynn, Skelly, & Mead, 1998; Suplee, 2005; Thornton, 1997; Winickoff et al., 2010).

### 3.2 Characteristics of all included trials

The details of participants, intervention, comparison group, outcome measures, main findings and BCTs identified are described in Supplementary Table 2. The majority of studies (25 trials) were conducted in the USA,(Allen et al., 2016; Brandon et al., 2012; Cummins et al., 2016; El-Mohandes et al., 2011; Ershoff et al., 1995; Forray et al., 2016; Forray & Waters, 2015; Kendrick et al., 1995; Kientz & Kupperschmidt, 2005; Levine et al., 2016; Lillington et al., 1995; McBride et al., 1999; McBride et al., 2004; Morasco et al., 2006; Mullen et al., 2001; Pbert et al., 2004; Petersen et al., 1992; Pollak et al., 2016; Reitzel et al., 2010; Ruger et al., 2008; Secker-Walker et al., 1995; Secker-Walker et al., 1998; Strecher et al., 2000; Suplee, 2005; Winickoff, Healey, Regan, et al., 2010) two in Canada,(Edwards & Sims-Jones, 1997; Johnson et al., 2000) two in the UK,(Hajek et al., 2001; Thornton, 1997) and three elsewhere in Europe (Germany, Spain and Poland)(Hannover et al., 2009; Jimenez-Muro et al., 2013; Polanska et al., 2004); all notably high income countries. Less than a third of interventions targeted lower socioeconomic groups or ethnic minorities (El-Mohandes et al., 2011; Kientz & Kupperschmidt, 2005; Levine et al., 2016; Lillington et al., 1995; Morasco et al., 2006; Reitzel et al., 2010; Ruger et al., 2008; Secker-Walker et al., 1998; Suplee, 2005; Thornton, 1997).

Interventions were heterogeneous, differing in intervention content, mode of delivery, and population. Thirteen trials included mixed populations of pregnant smokers and quitters (Cummins et al., 2016; Edwards & Sims-Jones, 1997; El-Mohandes et al., 2011; Hajek et al., 2001; Kendrick et al., 1995; Lillington et al., 1995; McBride et al., 1999; Morasco et al., 2006; Pbert et al., 2004; Petersen et al., 1992; Polanska et al., 2004; Ruger et al., 2008; Strecher et al., 2000); eleven included pregnant quitters (Brandon et al., 2012; Ershoff et al., 1995; Forray et al., 2016; Forray & Waters, 2015; Kientz & Kupperschmidt, 2005; Levine et al., 2016; Pollak et al., 2016; Reitzel et al., 2010; Secker-Walker et al., 1995; Secker-Walker et al., 1998; Suplee, 2005); two included mixed populations of postpartum smokers and quitters (Hannover et al., 2009; Jimenez-Muro et al., 2013); two included postpartum quitters (Allen et al., 2016; Johnson et al., 2000); three included pregnant women and partners (McBride et al., 2004; Mullen et al., 2001; Thornton, 1997); and one included postpartum women and partners (Winickoff et al., 2010). Interventions in most trials (*n* = 21) consisted of face-to-face or telephone advice (counselling, cognitive behavioural therapy, motivational interviewing) plus additional support (mainly written self-help materials), with the remainder using: counselling alone (El-Mohandes et al., 2011; Secker-Walker et al., 1995; Secker-Walker et al., 1998); self-help materials alone (Brandon et al., 2012; Mullen et al., 2001; Petersen et al., 1992; Strecher et al., 2000); oral progesterone (Allen et al., 2016; Forray et al., 2016); mobile phone alerts (Forray & Waters, 2015); incentives plus counselling and supporting materials (Lillington et al., 1995). Interventions were delivered by a wide range of clinical staff, pregnancy specialists, counsellors or researchers in either home or clinic settings. The intensity, duration, and time between intervention sessions varied widely from the provision of a single session to a maximum of 14 sessions provided up to 9 months postpartum. Only two trials followed up participants beyond 12 months postpartum (Hannover et al., 2009; Secker-Walker et al., 1995). We found no clear pattern as to which modes of delivery and timings of intervention contact would be best used to deliver intervention components most effectively.

Smoking status was validated in at least a sample of the population at a particular time point in 26 trials, but six trials only used self-report data (Edwards & Sims-Jones, 1997; Forray & Waters, 2015; Hannover et al., 2009; Kientz & Kupperschmidt, 2005; Polanska et al., 2004; Thornton, 1997). Many trials reported some fidelity and/or acceptability issues, and we considered 10 trials as having reported major intervention fidelity and/or acceptability concerns,(Edwards & Sims-Jones, 1997; Hajek et al., 2001; Hannover et al., 2009; Johnson et al., 2000; Kendrick et al., 1995; Kientz & Kupperschmidt, 2005; McBride et al., 1999; Morasco et al., 2006; Pbert et al., 2004; Thornton, 1997) which may have influenced intervention effectiveness. These trials highlighted issues of low recruitment, low level of intervention implementation, low level of uptake of the intervention, or high dropout rates. There was no distinct pattern as to which specific components of interventions had been most affected by fidelity and/or acceptability, since this level of detail was often not reported. Some trials made no reference, or minor reference, to fidelity related issues suggesting there may also have been some underreporting of concerns relating to protocol adherence, acceptability, or compliance.

### 3.3 Characteristics of long-term effective trials

We found six long-term effective trials demonstrating a statistically significant difference in smoking abstinence between the intervention and control groups at six months postpartum or later (Brandon et al., 2012; Cummins et al., 2016; Hannover et al., 2009; McBride et al., 1999; Mullen et al., 2001; Reitzel et al., 2010). Five of these trials were conducted in the USA and one in Germany (Hannover et al., 2009). Three trials targeted abstinent pregnant women,(Brandon et al., 2012; Mullen et al., 2001; Reitzel et al., 2010) one of which also included partners,(Mullen et al., 2001) two trials targeted mixed populations of pregnant smokers and quitters (Cummins et al., 2016; McBride et al., 1999) and one trial included postpartum smokers and quitters (Hannover et al., 2009). Four out of six interventions included an element of one-to-one counselling plus the provision of self-help materials, whilst two interventions (Brandon et al., 2012; Mullen et al., 2001) provided only posted self-help educational materials. Control groups received a less intensive intervention or usual care. All long-term effective interventions took place in home settings and were largely delivered remotely (telephone calls, posted or emailed materials) using a variety of intensities and durations. Where counselling was incorporated into the intervention, this was provided by specialist smoking cessation trained staff or by general trained counsellors. All but one of these trials (Hannover et al., 2009) reported some biochemical validation of outcome. Two long-term effective trials (Hannover et al., 2009; McBride et al., 1999) reported intervention fidelity and/or acceptability concerns: low adherence to motivational interviewing by trained counsellors (Hannover et al., 2009); or low levels of engaging women in smoking related issues in counselling calls (McBride et al., 1999) Had these issues not been present, it is possible that the intervention effect may have been stronger.

### 3.4 Behaviour change techniques in trials and those characterised as ‘promising’

BCTs targeting improvements to smoking behaviour, that we identified from intervention descriptions in our included trials, are summarised in Table 1. BCTs for each individual trial are shown in Supplementary Table 2 (definitions of BCT codes are in Table 1). A total of 45 of the 93 BCTs from the BCTTv1 were identified. The BCTTv1 organises 93 individual BCTs into 16 different groups according to similarity of individual BCT components (Michie et al., 2013). All groups were present in our included trials, with the exception of the group related to ‘scheduled consequences’ (concerning punishments and costs versus rewards). The number of BCTs identified in individual interventions ranged from one to 19, with an average of 7.7 BCTs per intervention. Focusing only on long-term effective interventions,(Brandon et al., 2012; Cummins et al., 2016; Hannover et al., 2009; McBride et al., 1999; Mullen et al., 2001; Reitzel et al., 2010) the number of BCTs identified ranged from five to 19 with an average of 11 BCTs per intervention. The most prevalent BCTs in all included trials (coded in ≥25% interventions) were: ‘problem solving’ (n=27); ‘information about social and environmental consequences' (n=27); ‘social support (unspecified)’ (*n* = 25); ‘information about health consequences' (*n* = 18); ‘credible source’ (*n* = 17); ‘instruction on how to perform a behaviour’ (*n* = 11); and ‘reduce negative emotions' (*n* = 9). For example, many trials gave information to participants on the health effects of smoking, or effects of exposure to environmental tobacco smoke.

We characterised six BCTs as ‘promising’, or most likely to enhance effectiveness of interventions to maintain smoking abstinence. The BCTs which we identified as ‘promising’ were: ‘problem solving’ in all (i.e., 100%) of long-term effective interventions; ‘information about health consequences’ (100%); ‘information about social and environmental consequences’ (100%); ‘social support’ (100%); ‘reduce negative emotions’ (50%); and ‘instruction on how to perform a behaviour’ (33%). If we were to remove the two trials (Hannover et al., 2009; McBride et al., 1999) which reported fidelity and/or acceptability concerns in our analysis, these six BCTs would still meet our definition of ‘promising’, or most likely to be effective.

**Table 1:** Frequency of BCTs identified in smoking relapse interventions.

BCT code	BCT label	BCT in all studies; n (%); Max *n* = 32	BCT in ‘long-term effective’# studies; n (%); Max *n* = 6
1.1	Goal setting (behavior)	5 (16)	1 (17)
1.2	Problem solving	27 (84)	6 (100) *
1.4	Action planning	1 (3)	0 (0)
1.5	Review behavior goal(s)	4 (13)	0 (0)
1.6	Discrepancy between current behavior and goal	1 (3)	1 (17)
1.7	Review outcome goal(s)	2 (6)	0 (0)
1.8	Behavioural contract	2 (6)	0 (0)
1.9	Commitment	3 (9)	1 (17)
2.2	Feedback on behavior	1 (3)	0 (0)
2.3	Self-monitoring of behavior	3 (9)	0 (0)
2.6	Biofeedback	4 (13)	0 (0)
3.1	Social support (unspecified)	25 (78)	6 (100) *
3.2	Social support (practical)	3 (9)	1 (17)
3.3	Social support (emotional)	3 (9)	1 (17)
4.1	Instruction on how to perform a behavior	11 (34)	2 (33) *
4.2	Information about antecedents	2 (6)	0 (0)
4.3	Re-attribution	1 (3)	1 (17)
5.1	Information about health consequences	18 (56)	6 (100) *
5.3	Information about social and environmental consequences	27 (84)	6 (100) *
5.6	Information about emotional consequences	1 (3)	1 (17)
6.1	Demonstration of the behavior	2 (6)	1 (17)
6.2	Social comparison	4 (13)	3 (50)
7.1	Prompts/cues	4 (13)	1 (17)
8.1	Behavioural practice/ rehearsal	2 (6)	0 (0)
8.2	Behavior substitution	5 (16)	2 (33)
9.1	Credible source	17 (53)	1 (17)
9.2	Pros and cons	7 (22)	3 (50)
9.3	Comparative imagining of future outcomes	1 (3)	0 (0)
10.1	Material incentive (behavior)	1 (3)	0 (0)
10.2	Material reward (behavior)	1 (3)	0 (0)
10.3	Non-specific reward	1 (3)	0 (0)
10.4	Social reward	6 (19)	2 (33)
10.7	Self-incentive	1 (3)	0 (0)
11.1	Pharmacological support	3 (9)	1 (17)
11.2	Reduce negative emotions	9 (28)	3 (50) *
12.1	Restructuring the physical environment	2 (6)	1 (17)
12.2	Restructuring the social environment	2 (6)	1 (17)
12.3	Avoidance/reducing exposure to cues for the behavior	4 (13)	2 (33)
12.5	Adding objects to the environment	4 (13)	2 (33)
13.1	Identification of self as role model	3 (9)	2 (33)
13.2	Framing/reframing	7 (22)	2 (33)
13.5	Identity associated with changed behavior	2 (6)	2 (33)
15.1	Verbal persuasion about capability	7 (22)	1 (17)
15.3	Focus on past success	1 (3)	1 (17)
16.2	Imaginary reward	3 (9)	2 (33)

# p < .05 in intervention vs control at ≥6 months postpartum; *BCTs identified as ‘promising’.

## 4 Discussion

This review is the first study, to our knowledge, to identify BCTs most promising for preventing postpartum smoking relapse. We identified a wide range of BCTs, finding an average of 7.7 BCTs per intervention for all included trials, and an average of 11 BCTs per intervention for long-term effective trials. There is a possibility that the effectiveness of interventions might be improved by incorporating more BCTs, implying more ‘complex’ interventions. We found six ‘promising’ BCTs, which were both frequently occurring and present in interventions that demonstrated long-term effectiveness of sustained smoking abstinence postpartum. These were: ‘problem solving’ which included advice on smoking relapse prevention; ‘information about health consequences’; ‘information about social and environmental consequences’ such as the effects of second-hand smoke; ‘social support’ from a partner or other supporter; ‘reduce negative emotions’ including stress management; and ‘instruction on how to perform a behaviour’ including skills training. Previous studies have identified effective BCTs using the smoking specific taxonomy (West et al., 2010) for smoking cessation in pregnancy (Lorencatto et al., 2012); and cessation for smokers in general (Lorencatto et al., 2013; West et al., 2010). This taxonomy has largely been superseded by the BCTTv1 designed for use in all behaviour change interventions (Michie et al., 2013). More recently, Campbell (Campbell et al., 2018) identified BCTs for smoking cessation in pregnancy based on combining the smoking specific taxonomy with the BCTTv1. However, they only included trials from a single Cochrane review (Chamberlain et al., 2017). Our results concur with those of Campbell et al. in finding the following BCTs to be potentially effective, based on their presence in two or more effective interventions: ‘information about consequences’; ‘problem solving’; ‘social support’ and ‘reduce negative emotions’. In total their review found 23 potentially effective techniques, but did not find the BCT ‘instruction on how to perform a behaviour’ to be present in effective interventions. However, providing advice on stop smoking medication and changing routine, have been identified as important BCTs for general smokers (smokers not necessarily pregnant or postpartum) (Lorencatto et al., 2013; West et al., 2010). Differences in our findings may be since these previous reviews were focused on smoking cessation, whilst we aimed to find BCTs effective for relapse prevention in the postpartum period.

Pregnant and postpartum smokers tend to be of a lower socioeconomic status, less well educated and have lower levels of support (Chamberlain et al., 2017; Orton et al., 2018). Future interventions and their BCT components need to be tailored to address the specific needs and cultural identity of these high-risk groups. Women who return to smoking postpartum may be less aware of the detrimental effects to the health of the baby caused by their return to smoking (Orton et al., 2018). This suggests the BCTs we identified concerning provision of ‘information about health consequences’ and ‘information about social and environmental consequences’, would be fundamental to the success of an intervention to prevent postpartum smoking relapse. Having greater social or partner support is known to be a key facilitator to prevent relapse (Flemming et al., 2015; Notley et al., 2015; Orton et al., 2018) and our finding of the importance in providing enhanced social support is consistent with this. Only four trials included in this review specifically engaged with partner groups. A review of postpartum relapse strategies concluded that more programmes should include a woman's partner and her wider social support network (Fang et al., 2004). We found BCTs prompting problem solving and providing guidance on how to maintain smoking abstinence to be ‘promising’, corresponding well with literature finding predictors for relapse include having a lower confidence to remain abstinent postpartum (Orton et al., 2018). Other predictors for relapse are experiencing higher levels of stress, depression and anxiety, and women who smoke are more likely to use substances in response to these circumstances (Chamberlain et al., 2017; Orton et al., 2018). Our finding of the importance in including the BCT to ‘reduce negative emotions’ might therefore improve the chances of success of an intervention to maintain smoking abstinence.

Many factors influence the effectiveness of BCTs within interventions in addition to the content; including intervention fidelity, acceptability, the mode of delivery, setting, duration, intensity and characteristics of the provider (Lorencatto et al., 2012; Michie, Fixsen, Grimshaw, & Eccles, 2009). Considering all of our included trials, we found no clear pattern relating these characteristics to the effectiveness of BCTs although, as noted by others, descriptions of intervention characteristics and fidelity related issues were limited in published papers (Michie et al., 2009). Long-term effective interventions that we identified all provided self-help support, and four out of six long-term effective trials combined self-help with counselling. For continued smoking abstinence postpartum, Chamberlain (Chamberlain et al., 2017) found some evidence for positive effects of health education, counselling and incentives. For smoking cessation in pregnancy, self-help interventions, including those delivered digitally (by computer or as text messages), have been found to be effective. (Griffiths et al., 2018; Naughton, Prevost, & Sutton, 2008). Self-help provided as written or electronic support, or as part of counselling, may be favourable modes of delivery for BCTs and worthy of further research. Long-term effective interventions included in this study were delivered in home settings and mostly delivered remotely (telephone calls, posted or emailed materials). This suggests effective interventions might be delivered relatively cost-effectively. However, fidelity issues relating to protocol adherence, poor acceptability or participant compliance were evident in two of these trials,(Hannover et al., 2009; McBride et al., 1999) which may have limited intervention effectiveness. Future interventions need to consider issues of implementation and acceptability in this population, who may be challenging to engage.

Potential limitations to this review included the heterogeneity of participants, types of interventions and outcomes. Therefore, as in similar BCT content reviews,(Campbell et al., 2018; Lorencatto et al., 2012) we did not attempt meta-analysis. As noted elsewhere in the literature,(Michie et al., 2009) the level of detail needed for BCT coding was often not present in published intervention descriptions. In practice, interventions may have used more BCTs than those reported. We did not contact study authors, but did address this by taking an inclusive approach in our coding, including BCTs coded as probably present (+) in addition to those coded as definitely (++) present (www.bct-taxonomy.com). We included a second independent coder for 10% of data extraction and involved a third reviewer where necessary to resolve discrepancies and to ensure any relevant BCTs had been correctly identified. Unfortunately we were unable to fully duplicate screening and data extraction due to limited resources. We did not assess risk of bias, as our main aim was to identify any potentially relevant BCTs, but we did describe any reported fidelity and/or acceptability issues, to consider how BCTs might be best delivered. As in other reviews (Campbell et al., 2018) we did not compare BCTs within effective and non-effective interventions. No trials reported a negative intervention effect and using this approach may have limited the number of potentially relevant BCTs. Measures of study effectiveness are also acknowledged to be ambiguous, since statistical significance is dependent on a wide range of factors, such as sample size (Peters, de Bruin, & Crutzen, 2015). We cannot say with certainty that the presence of individual BCTs, demonstrates any causal relationship with trial outcome, but their repeated presence across long-term effective trials suggests they may be more promising approaches for future intervention content.

Not all trials validated smoking abstinence biochemically, although a Cochrane review reported similar effect sizes in pregnancy trials which were validated and trials which were not (Chamberlain et al., 2017). All trials were conducted in high-income countries with the majority conducted in the USA; which may mean findings would not be generalizable to other countries or contexts. Smoking in pregnancy is reducing in higher income countries but increasing in low and middle-income countries (Chamberlain et al., 2017; Coleman et al., 2015) and no such countries met the inclusion criteria for this review. However, this may be since we excluded articles not published in English. The majority of included trials were targeted to general populations of pregnant or postpartum women, rather than populations of ethnic minorities or lower socioeconomic groups, where smoking prevalence is higher. Further research with these specific groups would be beneficial and might identify further relevant BCTs, such as BCTs concerning identity specific to different populations. Difficulty in adapting to a new mothering identity has been identified as a key factor in prompting return to smoking postpartum (Flemming et al., 2015; Notley et al., 2015). We did not identify any trials that focused on nicotine replacement therapy (NRT) or were recent enough to include more novel approaches such as e-cigarettes. Evidence suggests that e-cigarettes are significantly less harmful than smoking tobacco and many smokers use e-cigarettes as an aid to quit (DoH, 2017; McNeill, Brose, Calder, Bauld, & Robson, 2018). There is some evidence that for pharmacological interventions for smoking cessation in pregnancy, behavioural support in combination with NRT might be helpful (Coleman et al., 2015).

Strengths to this review included the systematic approach to identifying relevant interventions and the application of the most recent behaviour change technique taxonomy (Michie et al., 2013). We characterised BCTs as ‘promising’ if present in long-term effective interventions and where frequency in all included trials (regardless of effectiveness) was high. We were therefore able to consider ‘promising’ BCTs in light of their overall frequency in interventions relevant to this population, giving an indication of the ease by which these BCTs might be incorporated into interventions, their suitability to the setting, feasibility and acceptability. This is one of the first systematic reviews of BCTs specifically to prevent return to smoking postpartum and these results further our understanding of what behavioural support might be effective. Future research could compare interventions with different combinations of BCTs, or could compare BCTs in intervention and control groups. The identification and testing of BCTs in future postpartum relapse prevention interventions in a more diverse range of countries, with more diverse populations, and incorporating more novel components such as e-cigarettes would be beneficial to research and future practice. Identifying six specific BCTs as promising components for postpartum smoking relapse prevention highlights the need to customise support for this particular population. Further research by our team will explore these BCT components through qualitative research with postpartum ex-smokers in order to develop a prototype intervention for maintenance of smoking abstinence postpartum (MRC PHIND grant ref.: MR/P016944/1).

## 5 Conclusions

We identified six promising BCTs to prevent postpartum smoking relapse based on a structured systematic review of published available evidence. Future interventions should consider the inclusion of BCTs addressing problem solving and how to maintain abstinent behaviour, information on health and other consequences of smoking, reducing negative emotions and improving the likelihood of smoking abstinence through the provision of social support.

The following are the supplementary data related to this article.

Supplementary table 1 Supplementary table 1

Supplementary table 2 Supplementary table 2

## Funding

This work was supported by the Medical Research Council (PHIND grant ref.: MR/P016944/1).

## Declaration of interests

None declared.
