# Supplementary material for: A systematic review of behaviour change techniques within interventions to prevent return to smoking postpartum
Source: Addict Behav. 2019 May;92:236–43. doi: 10.1016/j.addbeh.2018.12.031 (PMC6518963; doi:10.1016/j.addbeh.2018.12.031)
Supplement: Supplementary table 1 [file mmc1.pdf]

Supplementary Table 1. References to included trials

Main trial	Trial article references
Allen 2016	Allen SS, Allen AM, Lunos S, Tosun N. Progesterone and postpartum smoking relapse: A pilot double-blind placebo-controlled randomized trial. Nicotine Tob Res. 2016;18(11):2145-2153.
Brandon 2012	*Brandon TH, Simmons VN, Meade CD, et al. Self-help booklets for preventing postpartum smoking relapse: a randomized trial. Am J Public Health. 2012;102(11):2109-2115.<br><br>Correa JB, Simmons VN, Sutton SK, Meltzer LR, Brandon TH. A content analysis of attributions for resuming smoking or maintaining abstinence in the post-partum period. Matern Child Health J. 2015;19(3):664-674.<br><br>Lopez E, Simmons V, Meade C, Quinn G, Pedraza J, Brandon T. The elusive pregnant ex-smoker: lessons from recruitment for a clinical trial. Society for Research on Nicotine and Tobacco 11th Annual Meeting, Prague. 2005.<br><br>Lopez EN, Simmons VN, Quinn GP, Meade CD, Chirikos TN, Brandon TH. Clinical trials and tribulations: lessons learned from recruiting pregnant ex-smokers for relapse prevention. Nicotine Tob Res. 2008;10(1):87-96.<br><br>Quinn G, Ellison BB, Meade C, et al. Adapting smoking relapse-prevention materials for pregnant and postpartum women: formative research. Matern Child Health J. 2006;10(3):235-245.<br><br>Simmons VN, Cruz LM, Brandon TH, Quinn GP. Translation and adaptation of smoking relapse-prevention materials for pregnant and postpartum Hispanic women. J Health Commun. 2011;16(1):90-107.<br><br>Simmons VN, Lopez Khoury E, Segall Koltz EJ, et al. Preventing smoking relapse among pregnant and postpartum women: a randomized clinical trial. Joint conference of SRNT and SRNT-E, Dublin. 2009:POS2-16.<br><br>Simmons VN, Sutton SK, Quinn GP, Meade CD, Brandon TH. Prepartum and postpartum predictors of smoking. Nicotine Tob Res. 2014;16(4):461-468.
Cummins 2016	Cummins SE, Tedeschi GJ, Anderson CM, Quinlan-Downs R, Harris P, Zhu S-H. Telephone counselling for pregnant smokers: essential elements. J Smok Cessat. 2007;2(2):36-46.<br><br>*Cummins SE, Tedeschi GJ, Anderson CM, Zhu SH. Telephone intervention for pregnant smokers: a randomized controlled trial. Am J Prev Med. 2016;51(3):318-326.
DC-HOPE (District of Columbia Healthy Outcomes of Pregnancy Education) El-Mohandes 2011	Backonja U, Robledo CA, Wallace ME, Flores KF, Kiely M. Reproductive health knowledge among African American women enrolled in a clinic-based randomized controlled trial to reduce psychosocial and behavioral risk: Project DC-HOPE. Women's Health Issues. 2016;26(4):442-451.<br><br>Blake S, El-Mohandes A, Schwartz D, El-Khorazaty N, Gantz M, Joseph J. Promoting smoking cessation during pregnancy and preventing postpartum relapse. Pediatric Academic Societies Annual Meeting; 2005; Washington, DC.<br><br>Blake S, Joseph J, Schwartz D, El-Khorazaty N, Gantz M, El-Mohandes A. Preventing

	prenatal and postpartum environmental tobacco smoke (ETS) exposure. Pediatric Academic Societies Annual Meeting; 2005; Washington, DC.<br><br>Blake SM, Murray KD, El-Khorazaty MN, et al. Environmental tobacco smoke avoidance among pregnant African-American nonsmokers. Am J Prev Med. 2009;36(3):225-234.<br><br>El-Khorazaty MN, Johnson AA, Kiely M, et al. Recruitment and retention of low-income minority women in a behavioral intervention to reduce smoking, depression, and intimate partner violence during pregnancy. BMC Public Health. 2007;7:233.<br><br>*El-Mohandes AA, El-Khorazaty MN, Kiely M, Gantz MG. Smoking cessation and relapse among pregnant African-American smokers in Washington, DC. Matern Child Health J. 2011;15 Suppl 1:S96-105.<br><br>El-Mohandes AA, Kiely M, Blake SM, Gantz MG, El-Khorazaty MN. An intervention to reduce environmental tobacco smoke exposure improves pregnancy outcomes. Pediatrics. 2010;125(4):721-728.<br><br>El-Mohandes AAE. A psycho-behavioral intervention on African American pregnant women with a history of intimate partner violence (IPV) improves birth weight distribution of their newborns. Pediatric Academic Societies Annual Meeting; 2005; San Francisco, CA.<br><br>El-Mohandes AAE. An integrated psycho-behavioral intervention during pregnancy has significant effects in reducing risks during the post-partum period in African-American women. Pediatric Academic Societies Annual Meeting; 2005; Washington, DC.<br><br>El-Mohandes AAE, Kiely M, Gantz MG, El-Khorazaty N. A multiple risk factor behavioral intervention reduces environmental tobacco smoke exposure. Pediatric Academic Societies Annual Meeting; 2007; Toronto, Canada.<br><br>El-Mohandes AAE, Kiely M, Gantz MG, El-Khorazaty MN. Very preterm birth is reduced in women receiving an integrated behavioral intervention: a randomized controlled trial. Matern Child Health J. 2011;15(1):19-28.<br><br>El-Mohandes AAE, Kiely M, Joseph JG, et al. An integrated intervention in pregnant African Americans reduces postpartum risk: a randomized trial. Obstet Gynecol. 2008;112(3):611-620.<br><br>El-Mohandes AE. An integrated behavioral intervention reduces rates of moderate and extreme prematurity in African American (AA) mothers with a history of smoking during pregnancy. Pediatric Academic Societies Annual Meeting; 2006; San Francisco, CA.<br><br>Joseph J. Randomized trial to reduce 4 behaviors linked to adverse pregnancy outcomes among 1048 inner-city African American women. Pediatric Academic Societies Annual Meeting; 2005; Washington, DC.<br><br>Joseph JG, El-Mohandes AA, Kiely M, et al. Reducing psychosocial and behavioral risk factors: results of a multisite intervention randomized trial of high-risk pregnant African American women. Am J Pub Health. 2009;99(6):1053-1061.<br><br>Katz KS, Blake SM, Milligan RA, et al. The design, implementation and acceptability of

	an integrated intervention to address multiple behavioral and psychosocial risk factors among pregnant African American women. BMC Pregnancy Childbirth. 2008;8:22.<br><br>Kiely M, El-Khorazaty MN, El-Mohandes AAE. Depression and smoking during pregnancy impact the efficacy of an integral behavioral intervention to resolve risks. Pediatric Academic Societies Annual Meeting; 2007; Toronto, Canada.<br><br>Subramanian S, Katz KS, Rodan M, et al. An integrated randomized intervention to reduce behavioral and psychosocial risks: pregnancy and neonatal outcomes. Matern Child Health J. 2012;16(3):545-554.<br><br>Tan S, Courtney LP, El-Mohandes AA, et al. Relationships between self-reported smoking, household environmental tobacco smoke exposure and depressive symptoms in a pregnant minority population. Matern Child Health J. 2011;15 Suppl 1:S65-74.
Edwards 1997	Edwards N, Sims-Jones N. Smoking and smoking relapse during pregnancy and postpartum: results of a qualitative study. Birth. 1998;25(2):94-100.<br><br>*Edwards NC, Sims-Jones N. A randomized controlled trial of alternative approaches to community follow-up for postpartum women. Can J Public Health. 1997;88(2):123-128.
Ershoff 1995	Ershoff DH, Mullen PD, Quinn VP. A randomized trial of a serialized self-help smoking cessation program for pregnant women in an HMO. Am J Pub Health. 1989;79(2):182-187.<br><br>*Ershoff DH, Quinn VP, Mullen PD. Relapse prevention among women who stop smoking early in pregnancy: a randomized clinical trial of a self-help intervention. Am J Prev Med. 1995;11(3):178-184.<br><br>Ershoff DH, Quinn VP, Mullen PD, Lairson DR. Pregnancy and medical cost outcomes of a self-help prenatal smoking cessation program in a HMO. Public Health Rep. 1990;105(4):340-347.<br><br>Mullen PD, Carbonari JP, Tabak ER, Glenday MC. Improving disclosure of smoking by pregnant women. Am J Obstet Gynecol. 1991;165(2):409-413.<br><br>Mullen PD, Quinn VP, Ershoff DH. Maintenance of nonsmoking postpartum by women who stopped smoking during pregnancy. Am J Public Health. 1990;80(8):992-994.<br><br>Mullen PD, Richardson MA, Quinn VP, Ershoff DH. Postpartum return to smoking: who is at risk and when. Am J Health Promot. 1997;11(5):323-330.<br><br>Quinn VP, Mullen PD, Ershoff DH. Women who stop smoking spontaneously prior to prenatal care and predictors of relapse before delivery. Addict Behav. 1991;16(1-2):29-40.
Forray 2015	*Forray A, Waters A. Ecological momentary assessments and attentional bias modification for postpartum smoking. Neuropsychopharmacology. 2015;40:S590-S591.

	Foster DW, Waters AJ, Forray A. Perinatal attentional retraining intervention for smoking - A pilot study. Drug Alcohol Depend. 2015;156:e73.
Forray 2016	Forray A, Gilstad-Hayden K, Sofuoglu M, Yonkers K. Progesterone for postpartum smoking relapse prevention. Neuropsychopharmacology. 2016;41:S606.
Hajek 2001	Hajek P, West R, Lee A, et al. Randomized controlled trial of a midwife-delivered brief smoking cessation intervention in pregnancy. Addiction. 2001;96(3):485-494.
Hannover 2009	*Hannover W, Thyrian JR, Roske K, et al. Smoking cessation and relapse prevention for postpartum women: results from a randomized controlled trial at 6, 12, 18 and 24 months. Addict Behav. 2009;34(1):1-8.<br><br>Roske K, Hannover W, Grempler J, et al. Post-partum intention to resume smoking. Health Educ Res. 2006;21(3):386-392.<br><br>Roske K, Schumann A, Hannover W, et al. Postpartum smoking cessation and relapse prevention intervention: a structural equation modeling application to behavioral and non-behavioral outcomes of a randomized controlled trial. J Health Psychol. 2008;13(4):556-568.<br><br>Thyrian JR, Freyer-Adam J, Hannover W, et al. Adherence to the principles of motivational interviewing, clients' characteristics and behavior outcome in a smoking cessation and relapse prevention trial in women postpartum. Addict Behav. 2007;32(10):2297-2303.<br><br>Thyrian JR, Freyer-Adam J, Hannover W, et al. Population-based smoking cessation in women post partum: adherence to motivational interviewing in relation to client characteristics and behavioural outcomes. Midwifery. 2010;26(2):202-210.<br><br>Thyrian JR, Hannover W, Grempler J, Roske K, John U, Hapke U. An intervention to support postpartum women to quit smoking or remain smoke-free. J Midwifery Womens Health. 2006;51(1):45-50.<br><br>Thyrian JR, Hannover W, Roske K, Rumpf HJ, John U, Hapke U. Postpartum return to smoking: identifying different groups to tailor interventions. Addict Behav. 2006;31(10):1785-1796.
HOPP (Healthy Options for Pregnancy and Parenting) McBride 1999	Curry SJ, McBride C, Grothaus L, Lando H, Pirie P. Motivation for smoking cessation among pregnant women. Psychol Addict Behav. 2001;15(2):126-132.<br><br>Lando HA, Valanis BG, Lichtenstein E, et al. Promoting smoking abstinence in pregnant and postpartum patients: a comparison of 2 approaches. Am J Manag Care. 2001;7(7):685-693.<br><br>McBride CM, Curry SJ, Grothaus LC, Nelson JC, Lando H, Pirie PL. Partner smoking status and pregnant smoker's perceptions of support for and likelihood of smoking cessation. Health Psychol. 1998;17(1):63-69.<br><br>*McBride CM, Curry SJ, Lando HA, Pirie PL, Grothaus LC, Nelson JC. Prevention of relapse in women who quit smoking during pregnancy. Am J Public Health. 1999;89(5):706-711.

Jimenez-Muro 2013	Jimenez-Muro A, Nerin I, Samper P, et al. A proactive smoking cessation intervention in postpartum women. Midwifery. 2013;29(3):240-245.
Johnson 2000	Bottorff JL, Johnson JL, Irwin LG, Ratner PA. Narratives of smoking relapse: the stories of postpartum women. Res Nurs Health. 2000;23(2):126-134.<br>*Johnson JL, Ratner PA, Bottorff JL, Hall W, Dahinten S. Preventing smoking relapse in postpartum women. Nurs Res. 2000;49(1):44-52.<br>Ratner PA, Johnson JL, Bottorff JL. Smoking relapse and early weaning among postpartum women: is there an association? Birth. 1999;26(2):76-82.
KICCS (Kientz Interventions for Continued Cessation of Smoking)<br>Kientz 2005	Kientz E, Kupperschmidt B. KICCS: a successful strategy to promote smoking cessation in women during and post pregnancy. Okla Nurse. 2005;50(4):27-30.
Lillington 1995	Lillington L, Royce J, Novak D, Ruvalcaba M, Chlebowski R. Evaluation of a smoking cessation program for pregnant minority women. Cancer Pract. 1995;3(3):157-163.
McBride 2004	McBride CM, Baucom DH, Peterson BL, et al. Prenatal and postpartum smoking abstinence a partner-assisted approach. Am J Prev Med. 2004;27(3):232-238.
Morasco 2006	Dornelas EA, Magnavita J, Beazoglou T, et al. Efficacy and cost-effectiveness of a clinic-based counseling intervention tested in an ethnically diverse sample of pregnant smokers. Patient Educ Couns. 2006;64(1-3):342-349.<br>*Morasco BJ, Dornelas EA, Fischer EH, Oncken C, Lando HA. Spontaneous smoking cessation during pregnancy among ethnic minority women: a preliminary investigation. Addict Behav. 2006;31(2):203-210.
NEWS study (Newborns Excel Without Second hand Smoke)<br>Winickoff 2010	Winickoff JP, Healey EA, Regan S, et al. Using the postpartum hospital stay to address mothers' and fathers' smoking: The NEWS Study. Pediatrics. 2010;125(3):518.
PANDA (Parents and Newborns Developing and Adjusting)<br>Mullen 2001	DiClemente C, Dolan-Mullen P, Windsor R. The process of pregnancy smoking cessation: implications for interventions. Tob Control. 2000;9(Suppl 3):iii16-iii21.<br>Mullen PD, DiClemente C, Carbonari J, et al. Project PANDA maintenance of pre-natal smoking abstinence postpartum at 6 weeks and 3, 6, and 12 months. Ann Behav Med. 1997;19:130.<br>*Mullen PD, DiClemente CC, Bartholomew LK. Theory and context in project PANDA: a program to help postpartum women stay off cigarettes. In: Bartholomew LK, Parcel GS, Kok G, Gottlieb NH, eds. Intervention Mapping: A Process for Designing Theory- and Evidence-based Health Promotion Programs. Palo Alto, CA: Mayfield: McGraw-Hill Publishing Co.; 2001.<br>Sockrider MM, Hudmon KS, Addy R, Mullen PD. An exploratory study of control of smoking in the home to reduce infant exposure to environmental tobacco smoke.

	Nicotine Tob Res. 2003;5(6):901-910.<br><br>Stotts AL, DiClemente CC, Carbonari JP, Mullen PD. Postpartum return to smoking: staging a "suspended" behavior. Health Psychol. 2000;19(4):324-332.
Peterson 1992	Petersen L, Handel J, Kotch J, Podedworny T, Rosen A. Smoking reduction during pregnancy by a program of self-help and clinical support. Obstet Gynecol. 1992;79:924-930.
Polanska 2004	Polanska K, Hanke W, Sobala W. Characteristic of the smoking habit among pregnant women on the base of the test "Why am I smoker?". Przegl Lek. 2005;62(10):1095-1098.<br><br>Polanska K, Hanke W, Sobala W. Smoking relapse one year after delivery among women who quit smoking during pregnancy. Int J Occup Med Environ Health. 2005;18(2):159-165.<br><br>*Polanska K, Hanke W, Sobala W, Lowe JB. Efficacy and effectiveness of the smoking cessation program for pregnant women. Int J Occup Med Environ Health. 2004;17(3):369-377.
Quit for Keeps<br>Stretcher 2000	Strecher VJ, Bishop KR, Bernhardt J, Thorp JM, Cheuvront B, Potts P. Quit for keeps: tailored smoking cessation guides for pregnancy and beyond. Tob Control. 2000;9 Suppl 3:Iii78-79.
Quit-for-Two<br>Pollak 2016	*Pollak KI, Fish LJ, Lyna P, et al. Efficacy of a nurse-delivered intervention to prevent and delay postpartum return to smoking: The quit for two trial. Nicotine Tob Res. 2016;18(10):1960-1966.<br><br>Pollak KI, Fish LJ, Lyna P, Peterson BL, Swamy GK, Levine MD. Predictors of pregnant quitters' intention to return to smoking postpartum. Nicotine Tob Res. 2015;17(6):742-745.
QT (Quit Together)<br>Pbert 2004	Bonollo DP, Zapka JG, Stoddard AM, Ma Y, Pbert L, Ockene JK. Treating nicotine dependence during pregnancy and postpartum: understanding clinician knowledge and performance. Patient Educ Couns. 2002;48(3):265-274.<br><br>Ma Y, Goins KV, Pbert L, Ockene JK. Predictors of smoking cessation in pregnancy and maintenance postpartum in low-income women. Matern Child Health J. 2005;9(4):393-402.<br><br>*Pbert L, Ockene JK, Zapka J, et al. A community health center smoking-cessation intervention for pregnant and postpartum women. Am J Prev Med. 2004;26(5):377-385.<br><br>Zapka J, Goins KV, Pbert L, Ockene JK. Translating efficacy research to effectiveness studies in practice: lessons from research to promote smoking cessation in community health centers. Health Promot Pract. 2004;5(3):245-255.<br><br>Zapka JG, Pbert L, Stoddard AM, Ockene JK, Goins KV, Bonollo D. Smoking cessation counseling with pregnant and postpartum women: a survey of community health center providers. Am J Public Health. 2000;90(1):78-84.
Reitzel 2010	Businelle MS, Kendzor DE, Reitzel LR, et al. Pathways linking socioeconomic status

	and postpartum smoking relapse. Ann Behav Med. 2013;45(2):180-191.<br><br>Correa-Fernandez V, Ji L, Castro Y, et al. Mediators of the association of major depressive syndrome and anxiety syndrome with postpartum smoking relapse. J Consult Clin Psychol. 2012;80(4):636-648.<br><br>Heppner WL, Ji L, Reitzel LR, et al. The role of prepartum motivation in the maintenance of postpartum smoking abstinence. Health Psychol. 2011;30(6):736-745.<br><br>Kendzor DE, Businelle MS, Costello TJ, et al. Breast feeding is associated with postpartum smoking abstinence among women who quit smoking due to pregnancy. Nicotine Tob Res. 2010;12(10):983-988.<br><br>*Reitzel LR, Vidrine JI, Businelle MS, et al. Preventing postpartum smoking relapse among diverse low-income women: a randomized clinical trial. Nicotine Tob Res. 2010;12(4):326-335.
Ruger 2008	Ruger JP, Emmons KM, Kearney MH, Weinstein MC. Measuring the costs of outreach motivational interviewing for smoking cessation and relapse prevention among low-income pregnant women. BMC Pregnancy Childbirth. 2009;9(46) doi: 10.1186/1471-2393-9-46.<br><br>*Ruger JP, Weinstein MC, Hammond SK, Kearney MH, Emmons KM. Cost-effectiveness of motivational interviewing for smoking cessation and relapse prevention among low-income pregnant women: a randomized controlled trial. Value Health. 2008;11(2):191-198.
SCIP (Smoking Cessation in Pregnancy) Kendrick 1995	England LJ, Kendrick JS, Wilson HG, Merritt RK, Gargiullo PM, Zahniser SC. Effects of smoking reduction during pregnancy on the birth weight of term infants. Am J Epidemiol. 2001;154(8):694-701.<br><br>*Kendrick JS, Zahniser SC, Miller N, et al. Integrating smoking cessation into routine public prenatal care: the Smoking Cessation in Pregnancy project. Am J Public Health. 1995;85(2):217-222.<br><br>Spierto FW, Hannon W, Kendrick J, Bernert J, Pirkle J, Gargiullo P. Urinary cotinine levels in women enrolled in a smoking cessation study during and after pregnancy. J Smoking-Related Dis. 1994;5(2):65-76.
Secker-Walker 1995	Secker-Walker RH, Solomon LJ, Flynn BS, et al. Individualized smoking cessation counseling during prenatal and early postnatal care. Am J Obstet Gynecol. 1994;171(5):1347-1355.<br><br>*Secker-Walker RH, Solomon LJ, Flynn BS, et al. Smoking relapse prevention counseling during prenatal and early postnatal care. Am J Prev Med. 1995;11(2):86-93.
Secker-Walker 1998	Secker-Walker RH, Solomon LJ, Flynn BS, et al. Training obstetric and family practice residents to give smoking cessation advice during prenatal care. Am J Obstet Gynecol. 1992;166(5):1356-1363.<br><br>Secker-Walker RH, Solomon LJ, Flynn BS, Skelly JM, Mead PB. Reducing smoking

	during pregnancy and postpartum: physician's advice supported by individual counseling. Prev Med. 1998;27(3):422-430.<br><br>*Secker-Walker RH, Solomon LJ, Flynn BS, Skelly JM, Mead PB. Smoking relapse prevention during pregnancy. A trial of coordinated advice from physicians and individual counseling. Am J Prev Med. 1998;15(1):25-31.<br><br>Secker-Walker RH, Vacek PM, Flynn BS, Mead PB. Estimated gains in birth weight associated with reductions in smoking during pregnancy. J Reprod Med. 1998;43(11):967-974.<br><br>Secker-Walker RH, Vacek PM, Flynn BS, Mead PB. Smoking in pregnancy, exhaled carbon monoxide, and birth weight. Obstet Gynecol. 1997;89(5 Pt 1):648-653.<br><br>Solomon LJ, Secker-Walker RH, Skelly JM, Flynn BS. Stages of change in smoking during pregnancy in low-income women. J Behav Med. 1996;19(4):350-366.
STARTS (Strategies to Avoid Returning to Smoking) Levine 2016	Emery RL, Gregory MP, Grace JL, Levine MD. Prevalence and correlates of a lifetime cannabis use disorder among pregnant former tobacco smokers. Addict Behav. 2016;54:52-58.<br><br>Levine MD, Cheng Y, Marcus MD, Emery RL. Psychiatric disorders and gestational weight gain among women who quit smoking during pregnancy. J Psychosom Res. 2015;78(5):504-508.<br><br>*Levine MD, Cheng Y, Marcus MD, Kalarchian MA, Emery RL. Preventing postpartum smoking relapse: A randomized clinical trial. Jama Intern Med. 2016;176(4):443-452.<br><br>Levine MD, Marcus MD, Kalarchian MA, Cheng Y. Strategies to Avoid Returning to Smoking (STARTS): A randomized controlled trial of postpartum smoking relapse prevention interventions. Contemp Clin Trials. 2013;36(2):10.1016/j.cct.2013.1010.1002.
Suplee 2005	Suplee PD. The importance of providing smoking relapse counseling during the postpartum hospitalization. J Obstet Gynecol Neonatal Nurs. 2005;34(6):703-712.
The Rotunda Stop Smoking Programme Thornton 1997	*Thornton L. The Rotunda Stop Smoking Programme: Smoking and Pregnancy: Feasability and Effectiveness of a Smoking Intervention Programme Among Pregnant Women. Ireland: Department of Public Health, Eastern Health Board;1997.<br><br>Thornton L, Gogan C, McKenna P. The rotunda stop smoking programme. Irish J Med Sci. 1998;167(Suppl 9):28.

* Denotes main trial reference
