# Supplementary material for: A systematic review of behaviour change techniques within interventions to prevent return to smoking postpartum
Source: Addict Behav. 2019 May;92:236–43. doi: 10.1016/j.addbeh.2018.12.031 (PMC6518963; doi:10.1016/j.addbeh.2018.12.031)
Supplement: Supplementary table 2 [file mmc2.pdf]

Supplementary Table 2. Intervention characteristics

Trial, lead author, year	Population (no. and type of participants, country)	Intervention and comparison	Main outcome measures	Main findings (including fidelity)	BCTs present #
Allen 2016	Abstinent postpartum women (4 days postpartum)<br>Randomised: 24 IG; 22 CG<br>Analysed: 21 IG; 19 CG<br>USA	IG: Progesterone (400mg/d) for 4 weeks (supplied at research clinics or in participant homes)<br><br>CG: "Identical" placebo for 4 weeks	Biochemically validated 7-day point prevalence abstinence at 12 weeks postpartum; self-reported continuous abstinence. Also compliance; acceptability; safety	At week 4 postpartum, abstinence was 75% in IG and 68% in CG (NS)<br>Retention rate was 87% at week 12; clinic visit attendance was 80%; adherence was 68%; no significant differences in adverse events, depression or breastfeeding, self-reported satisfaction score	1.2++IC<br>3.1++IC<br>11.1++I
* Brandon 2012	Abstinent pregnant women<br>Randomised: 343 IG; 357 CG<br>Analysed: 245 IG; 259 CG<br>USA	IG: Ten self-help relapse prevention booklets, posted until 8 months postpartum<br><br>CG: Two existing smoking relapse booklets posted	Self-reported 7-day point prevalence abstinence, biochemically validated for a subsample of participants at 1, 8 and 12 months postpartum. Also Client Satisfaction Questionnaire; other questionnaires including Fagerstrom Test for Nicotine Dependence	At month 8 postpartum, abstinence was 70% for IG and 59% in CG (p=0.02), however significance was not maintained at 12 months post-partum overall, but post-hoc analysis showed significance was maintained for low-income women<br>Return questionnaire rates were 88%, 82% and 79% at 1, 8 and 12 months postpartum, no statistical difference between groups. IG reported greater satisfaction and perceived novelty of materials	1.2++IC<br>3.1++I<br>5.1++I<br>5.3++IC<br>9.1++IC
* Cummins 2016	Pregnant (<27 weeks); 97%	IG: Telephone counselling, posted brochure and self-	Self-reported 30-day abstinence in the	At the end of pregnancy, abstinence was 30% for IG and 20% in CG (p<0.001); at 2 months	1.2++I<br>1.9+I

	smokers or 3% recent quitters (quit within 2 weeks)<br>Randomised: 584 IG; 589 CG<br>Analysed: 351 IG; 409 CG<br>USA	help materials. Nine counselling sessions (1st call lasting 45 minutes, 5 shorter follow-up calls in pregnancy, 1 pre-birth call lasting 30 minutes, 2 follow-up calls 2 and 4 weeks postpartum). Counsellors were veteran quit line staff, trained on the pregnancy-specific protocol<br><br>CG: Posted self-help materials	third trimester, 90-d abstinence 2 months postpartum and 180-d abstinence at 6 months postpartum. Biochemically validated 7-d point prevalence abstinence at the third trimester evaluation. Also quit attempt rate and related questions to increase accuracy of self-report	postpartum 22% vs 15% (p<0.05); at 6 months postpartum 14% vs 8% (p<0.05). Cotinine corrected abstinence in the third trimester 36% vs 23% (p<0.05)<br>Counsellors received weekly supervision to adhere to the protocol, and debriefing was available by colleagues	3.1++I<br>3.2+I<br>3.3+I<br>4.1++I<br>4.3+I<br>5.1+I<br>5.3+IC<br>6.1++I<br>9.2++I<br>12.5+I<br>13.2+I<br>13.5++I<br>16.2++I
DC-HOPE (District of Columbia Healthy Outcomes of Pregnancy Education) El-Mohandes 2011	African-American women (predominantly low income) reporting smoking in the 6 months preceding pregnancy; 39% current smokers at baseline. Subset recruited as part of DC-HOPE trial<br>Randomised: 262 IG; 238 CG<br>Analysed: 196 IG; 188 CG<br>USA	IG: Tailored cognitive behavioural therapy session meetings (up to 8 prenatal and 2 postpartum, each of 35-40 minutes. Attendance for at least 4 sessions was deemed "adequate") using stages of change techniques to reduce smoking, reduce environmental smoke exposure, depression and intimate partner violence. Sessions were delivered by trained counsellors<br><br>CG: Usual care (meeting primary care providers as per standard clinic practice)	Self-reported biochemically validated point prevalence abstinence at 22-26 weeks gestation, 30-34 weeks gestation, 8-10 weeks postpartum. Also factors associated with smoking behaviours	For this sub-set of participants: at 8-10 weeks postpartum the intervention was associated with higher levels of smoking abstinence (NS at p=0.053). Overall, 34% of women were considered postpartum relapsers (smoking in the postpartum period but not at follow-up interview during pregnancy)<br>For the larger DC-HOPE trial of 1070 randomised, when comparing risk-specific changes between baseline and the postpartum period, postpartum resurgence in smoking rates were significantly higher in CG (p<0.001), and reduction in environmental tobacco smoke exposure significantly higher in IG (p=0.011), although the difference between groups in the postpartum period was not significant. Generally rates of adverse pregnancy and neonatal outcomes did not differ between arms. Retention rate was 79%. 54%	1.2++I<br>1.5+I<br>1.7+I<br>2.3++I<br>3.1++I<br>3.3+I<br>4.1++I<br>5.1++I<br>5.3+I<br>6.1+I<br>10.3+I<br>11.2+I<br>12.1+I<br>12.3++I<br>13.2+I

				women attended at least 4 intervention sessions Counsellors were trained with weekly supervision, periodic direct observation and review of audiotaped transcripts. Perceptions of study participation was reported for a subsample of 152 women. Session content was delivered as prescribed in 77% or more sessions. For intervention women, 93% had a positive view of their relationship with the counsellor and most found the session helpful. More women in the IG resolved some or all of their risk factors	
Edwards 1997	Primiparous women (gestation 35 weeks or greater); any smoking status<br>Randomised: Unclear; 793 overall on assessment of final eligibility<br>Analysed: 279 IG arm 1; 218 IG arm 2; 291 CG<br>Canada	3 arm trial:<br>IG 1: Telephone Visit group: Mailed information on infant care, safety & nutrition ~10 days after delivery, including invitation to parent-baby groups facilitated by public health nurses; plus telephone call by public health nurse 1-2 weeks after discharge. Call was individually tailored and included smoking environment; and maintaining abstinence if quit during pregnancy<br><br>IG 2: Clerk call group: Mailed information on infant care, safety & nutrition ~10 days after delivery, including invitation	Infant care behaviour scores at 3 months postpartum (Including maternal smoking). Also use of postnatal support services. Qualitative interviews undertaken with a subsample of women	At 3 months postpartum, percentage of mothers reporting smoking were 19.5% IG 1, 18.2% IG 2, 17.9% CG (NS). No significant differences in infant-care behaviours<br>Significantly more women in IG 1 attended the parent-baby group than women in IG 2 or CG ($p<0.05$)<br>Attendance of the parent-baby group was 20% or less<br>Qualitative transcripts were reviewed on an ongoing basis to assess interviewer technique. 5 women in the clerk call group were only asked questions about awareness and utilisation of community resources due to an administrative error and were excluded from the analysis	1.2++I<br>3.1++IC<br>5.3++IC<br>8.2++I<br>9.1+I

		to parent-baby groups facilitated by public health nurses; plus telephone call by a Health Department clerk at 5 weeks postpartum for reminder of parent-infant baby group<br><br>CG: Mailed information on infant care, safety & nutrition ~10 days after delivery, including invitation to parent-baby groups facilitated by public health nurses			
Ershoff 1995	Abstinent pregnant women (<18 weeks); quit since becoming pregnant. Part of a wider cessation trial- current smokers were assigned to a separate smoking cessation intervention<br>Randomised: 110 IG; 108 CG<br>Analysed: 87 IG; 84 CG<br>USA	IG: Eight booklets to teach strategies for cessation (booklets 1-4 given at the intervention start) and relapse prevention (booklets 5-8, mailed weekly over the next 4 weeks); plus pamphlets and health educator intervention as for the control group<br><br>CG: Two pamphlets on the hazards of smoking and importance of abstinence plus health educator conference (including cigarette smoking) and	Self-reported, biochemically validated, continuous abstinence through delivery. Also self-reported 7-day biochemically validated abstinence and smoking behaviour, and attitudinal measures at weeks 26 and 34 of pregnancy. Status of self-reported continuous abstinence at 6 months postpartum reported for women	At delivery, biochemically confirmed continuous abstinence for completers, was 83.9% for IG and 79.8% for CG (NS)<br>At 6 months postpartum, for baseline quitters, 38.3% reported abstinence in IG and 36.4% in CG (NS). From the subset of 134 participants, overall maintenance rate at 6 months was 37% and mean time to relapse was 111 days ± 6.1<br>83% of women reported reading at least one of the booklets. Of these ~60% reported reading all 8. Of women claiming abstinence at the time of the interview, 54% stated the booklets were either "very" or "somewhat" helpful	1.2++I<br>3.1++IC<br>4.1IC<br>5.1++IC<br>5.3++IC<br>9.1++IC<br>10.4++IC<br>11.2+I

		interview; plus one page tip sheet on behavioural techniques to avoid relapse	whose abstinence was verified before the 20th week of pregnancy continuing through delivery (n=134, 82% of the sample from this study, 18% from the wider cessation trial)		
Forray 2015	Abstinent pregnant women<br>Randomised: 6 IG; 5 CG<br>Analysed: 6 IG; 5 CG<br>USA	IG: Attentional retraining using smartphone alerts (4 times/day) in the last month of pregnancy and immediately postpartum. Participants responded to questions assessing subjective states, followed by attentional retraining using a visual probe task<br><br>CG: Attentional control using smartphone alerts. Participants responded to questions assessing subjective states, followed by attentional control	Attentional bias for smoking and self-reported craving and smoking status (up to 2 weeks postpartum)	Attentional bias towards smoking stimuli was more negative in IG (n=35 assessments, M= -52.6 ms, sd= 122) than CG (n=44 assessments, M=-18.5 ms, sd 146) (p<0.05)<br>For all women, craving significantly increased from pregnancy (M= 1.40, sd= 1.23) to postpartum (M= 2.28, sd= 2.27). 8 women reported smoking at least 1 cigarette during the study; and postpartum participants reported having smoked since the last assessment on 13% of assessments<br>Participants carried the smartphone for a mean of 20.2 days and completed 444 assessments (60% pregnancy, 40% postpartum); and 2.92 assessments/day	1.2++I<br>12.5+IC<br>13.2+I
Forray 2016	Pregnant women achieving abstinence by 32 weeks of gestation<br>Randomised: 41 overall	IG: Oral micronized progesterone (200mg twice daily) following delivery for 8 weeks<br><br>CG: Placebo (unclear) following delivery for 8	Self-reported, biochemically validated, 7-day point prevalence abstinence at 8 weeks postpartum. Also continuous	At week 8 postpartum, abstinence was twice as likely in IG vs CG (NS at p=0.26); continuous abstinence was 1.7 times more likely in IG (NS at p=0.47); and this group had a 28% delay in time to relapse vs CG (NS at p=0.45)<br>Median time to relapse for IG was 52 days vs 38 days for CG	11.1++I

	Analysed: unclear<br>USA	weeks	abstinence for weeks 1 to 8		
Hajek 2001	Pregnant women (in third month of pregnancy); 78% smokers or 22% recent quitters (quit within previous 3 months)<br>Randomised: Unclear; 1287 recruited overall<br>Analysed: 545 IG; 575 CG<br>UK	IG: Extended midwife advice, brief counselling, written materials, arrangements for continuing self-help support and feedback on CO levels. The intervention was tailored to motivation to quit and whether women were current smokers or recent quitters<br><br>CG: Midwife usual care and standard anti-smoking leaflets	Self-reported biochemically validated continuous abstinence ≥3 months prior to delivery; self-reported biochemically validated continuous abstinence from 3 months pre-delivery to 6 months post-delivery; self-reported biochemically validated point prevalence abstinence at delivery. Also questionnaires recording recall and ratings of intervention components; and baby birth weight	At delivery, point-prevalence abstinence for recent ex-smokers was 65% in IG and 53% in CG ($p<0.05$). No other significant differences between groups, although the intervention significantly increased the desire to stop smoking at the postnatal interview ($p<0.02$). At 6 months postpartum 23% in IG and 3% in CG were abstinent (NS)<br>Researchers were available to give refresher training to midwives throughout the study, however midwife skills were not checked. Recruitment was low and the extent to which midwives followed the intervention protocol was variable, with only handing out booklets and using the CO monitor to be implemented comprehensively. A quit date was only negotiated by 56% of midwives for current smokers, and the social support "buddy" system was largely not initiated. 65% of midwives said the intervention could not be undertaken in the time available. Midwives queried whether the midwife booking appointment, which is generally busy, was the optimal time for the intervention. Some expressed concern of straining relationships with patients (traditional approach to cut-down rather than quit)	1.2++I<br>1.8++I<br>2.6++I<br>3.1+I<br>3.2+I<br>5.1+I<br>5.3++I<br>9.1++I<br>13.2+I
* Hannover 2009	Postpartum women (approached at delivery); 53% smokers and 47% recent quitters (quit no longer	IG: Face-to-face motivational counselling 40 days postpartum at home and telephone counselling calls 4 and 12 weeks later- tailored 45 minute motivational interviews	At 6, 12, 18 and 24 months postpartum, unvalidated self-reported (telephone interview): i) continuous abstinence (had not	Point prevalence abstinence at 6 months was significantly different: 38% in IG and 29% in CG ($p=0.009$). For women not smoking at baseline, there were no significant differences between the groups in smoking abstinence. At 24 months, relapse was 77% in IG and 75% in CG. For smokers at baseline, 4 week point prevalence abstinence	1.2++IC<br>1.6++I<br>3.1++I<br>5.1++I<br>5.3++IC<br>5.6++I<br>6.2++I

	than 4 weeks prior to pregnancy)<br>Randomised: 438 IG; 433 CG<br>Analysed: 226 IG; 257 CG<br>Germany	based on Transtheoretical Model, a tailored self-help manual and brochures as for the control group. Sessions delivered by trained counsellors<br><br>CG: Usual care plus 2 self-help brochures (1 addressing the partner)	smoked in the 6 months since delivery, or for subsequent follow-ups had not smoked in the 6 months since the last assessment); ii) repeated 4 week point prevalence abstinence (within the last 4 weeks prior to interview). Also self-efficacy, decisional balance and predictors of return to smoking	was higher in IG at 6, 12 and 18 months (7% vs 1%, 7% vs 2%, and 9% vs 1% respectively). Sustained abstinence achieved statistical significance only at 6 months postpartum (3% vs 0%). Overall the intervention showed a significant effect on smoking status at 6 months (p=0.009) and higher self-efficacy but not at 1 year<br>Counsellors were trained and received weekly supervision to ensure protocol adherence. Motivational Interviewing Treatment Integrity Code was used to evaluate adherence to interviewing, with the majority rated as proficient to expert quality. In a sample of 84 recorded sessions for smoking cessation, only 38% showed good adherence to motivational interviewing. Relationship between adherence and intervention outcome at 6 months was not significant. In a sample of 163 recorded sessions for combined smoking cessation and relapse prevention, 49% of sessions showed good adherence to MI. The OR of receiving a good intervention session was 3.1 for non-smokers in comparison to daily smokers. The relationship between adherence and intervention outcome at 6 months was significant (p<0.05).<br>Drop out was relatively high	8.2++I<br>9.2++I<br>10.4++I<br>11.1++I<br>12.1++IC<br>12.2+I<br>12.3++I<br>13.1++I<br>13.5++I<br>15.1++I<br>15.3++I<br>16.2+I
* HOPP (Healthy Options for Pregnancy and Parenting)<br>McBride 1999	Pregnant women (approached at first prenatal visit); 56% current smokers or 44% recent quitters (had smoked in the 30 days	3 arm trial:<br>IG 1: Self-help booklet plus pre- and postpartum intervention (pre/post group). Included posted/emailed materials (personalised letter, relapse prevention kit) and	Self-reported 7-day prevalence abstinence at 28 weeks of pregnancy. 7-day prevalence abstinence at 8, 6 and 12 months postpartum.	Prevalence abstinence was significantly greater in IG 1, only at 8 weeks and 6 months postpartum. At 8 weeks, abstinence was 39% in IG 1, 35% in IG 2, and 30% in CG (p=0.02). At 6 months, abstinence was 33% in IG 1, 24% in IG 2, and 26% in CG (p=0.04)<br>Smoking cessation counsellors received 40 hours of training and followed a standardised protocol	1.2++I<br>3.1++I<br>(arm1)<br>5.1++IC<br>5.3++IC<br>6.2++I<br>8.2++IC<br>10.4++I

	before pregnancy but quit by baseline)<br>Randomised: Unclear; 1007 overall<br>Analysed: 897 participated and included in intention-to-treat analysis 306 IG arm 1; 294 IG arm 2; 297 CG<br>USA	telephone counselling delivered by smoking cessation counsellors who were ex-smokers who had been through pregnancy. Sessions occurred prepartum, 3 calls (1st, 2 weeks after booklet mailing, 2nd and 3rd followed at 1 month intervals, mean duration 8.5 ±6.3 minutes). Plus 3 calls within the first 4 months postpartum (1st, 4 weeks after delivery, 2nd and 3rd followed at 4-6 week intervals, mean duration 7.7 ± 6.5 minutes). Plus postpartum newsletters (3 mailed at 2, 6 and 12 weeks postpartum)<br><br>IG 2: Self-help booklet plus prepartum intervention. As for pre/post group, minus postpartum calls and postpartum newsletters<br><br>CG: Self-help booklet only	Validated samples were obtained from women reporting not smoking in the previous 7 days at 28 weeks of pregnancy, 6 and 12 month postpartum follow-ups. Use of intervention components was assessed at 8 weeks postpartum. Also motivation for cessation using the reasons for quitting scale; and partner smoking status and perceptions of support	(format for calls was open-ended). Counsellors discussed cases and reported concerns to investigators on a regular basis<br>Participant response rates were 92% at 28 weeks of pregnancy. Postpartum response rates were 91% at 8 weeks, 89% at 6 months, 87% at 12 months. More women in the intervention arms recalled receiving the booklet than in CG (96% vs 81%). Approximately half of these women reported reading the booklet, and of those, more than half reported following some of its suggestions. 91% of women in the intervention arms recalled receiving the relapse prevention kit; 46% reported reading this; of those, 58% reported following suggestions. Participation in the counselling calls for the intervention arms was high (92% for the 1st prepartum call, 86% 2nd, 78% third). For IG 1, 82% accepted at least 1 postpartum call, 93% recalled receiving newsletters and 60% reported following some of its suggestions<br>Problems identified that related to intervention delivery related to initial identification of the target population and acceptance of repeated calls. In a large percentage of completed calls, counsellors were unable to actively engage women in discussion of smoking related issues	(arm1)<br>11.2++IC<br>12.3++IC
Jimenez-Muro 2013	Postpartum women (approached at delivery); 57% smokers and 43%	IG: An information booklet (designed for the study) plus four telephone sessions based on motivational interviewing and relapse	Self-reported biochemically validated continuous abstinence at 3 months postpartum	At 3 months postpartum, for recent quitters, using an intention to treat analysis, biochemically validated continuous abstinence was 31% in IG; 23% in CG (NS). Only half the sample attended the visit at 3 months to confirm abstinence	1.2++I<br>3.1++I<br>5.1++IC<br>5.3++I

	recent quitters (quit at the beginning or during pregnancy)<br>Randomised: 205 IG; 207 CG<br>Analysed: 101 IG; 99 CG<br>Spain	prevention in weeks 3, 6, 9 and 12. Sessions lasted 15 minutes and were delivered by a trained counsellor<br><br>CG: An information booklet (designed for the study) plus two check calls in weeks 3 and 12. Sessions lasted 2 minutes	(had not smoked since delivery)	biochemically<br>In recent quitters with self-reported abstinence, the probability of remaining abstinent for 3, 6, 9 and 12 weeks for the IG was 92%, 91%, 88% and 74% respectively vs 63% at 3 weeks and 37% at 12 weeks in the CG (p<0.001)<br>For smokers, 91% of the IG said they would be ready to try to quit in the following 6 months vs 18% for the CG (p<0.001)<br>No differences between the IG and CG in numbers participating in phone contacts (~90%) or the third month visit to validate abstinence (~50%)	
Johnson 2000	Postpartum women (approached at delivery) who quit smoking in pregnancy, or to aid conception<br>Randomised: 125 IG; 126 CG<br>Analysed: 121 IG; 120 CG<br>Canada	IG: Tailored pamphlets and materials plus face-to-face in hospital counselling sessions at birth followed by 8 telephone counselling sessions in the three months postpartum. Sessions were delivered by nurses and held during the first month and biweekly during the second and third months. Contacts ranged from 1 to 20 minutes<br><br>CG: "usual care" which did not contain any information about the effects of smoking or prevention of smoking relapse	Self-reported biochemically validated continuous abstinence at 6 months post delivery (had not smoked since delivery). Also self-reported daily smoking; and questionnaires including self-efficacy (measured using the Smoking Abstinence Self-Efficacy Scale) and Fagerstrom Tolerance. Qualitative interviews undertaken with a subsample of women	At 6 months, continuous smoking abstinence was 38% in IG and 27% in CG (NS). Significantly more control (48%) than intervention (34%) participants reported daily smoking (p=0.03). There were no differences in reported self-efficacy<br>Nurses were hired and trained to deliver the intervention<br>Only 25% participants received all eight telephone contacts. "Fluctuation in delivery of the intervention may account for the weaker than anticipated effect"	1.2++I<br>3.1++I<br>1.5+I<br>4.1++I<br>5.1++I<br>5.3++I<br>7.1++I<br>8.1++I<br>9.1+I<br>13.2++I<br>15.1+I
KICCS (Kientz	Abstinent pregnant women	IG: Personalised, stage based support	Self-reported demographic data	At 6 weeks postpartum, abstinence was 83% in IG vs 60% in CG (NS)	3.1++I<br>4.1++I

Interventions for Continued Cessation of Smoking) Kientz 2005	(ceased smoking for 30 days by 36 weeks gestation); predominantly low income<br>Randomised: Unclear; 12 overall<br>Analysed: 11 overall<br>USA	(Transtheoretical Model of Change), initiated at 36 weeks gestation until 6 weeks postpartum, comprising reading material, candy, and a follow-up phone call<br><br>CG: Unclear	including smoking status and Decisional Balance Scale (pros and cons of smoking) at 36 weeks gestation and 6 weeks postpartum	For both groups the mean "con" score was higher than the "pro" score at 36 weeks gestation and 6 weeks postpartum<br>"A limitation of the study was the 'student study' was sometimes overlooked when clinic work and workload situations took priority"	5.1++I<br>5.3++I<br>7.1++I<br>9.3+I<br>12.5++I
Lillington 1995	Pregnant African American and Hispanic women of low income; 40.5% smokers or 59.5% recent quitters (stopped smoking in the past year)<br>Randomised: Unclear; 768 overall<br>Analysed: 155 IG; 400 CG<br>USA	IG: "Time for a Change: A Program for Healthy Moms and Babies", 15 minute one-to-one counselling session and self-help guide, including behaviour change strategies, booster postcard and incentives. Counselling provided by bilingual health educators<br><br>CG: "usual care" including printed information about the risks of smoking during pregnancy and a group quit-smoking message	Self-reported smoking cessation and relapse at end of pregnancy; self-reported biochemically validated abstinence and quit rates at 6 weeks postpartum. Also participant views of intervention	At 6 weeks postpartum self-reported abstinence was 79% in IG and 62% in CG (p<0.01). When adjusted by validation analysis, relapse rates were 36% in IG and 49% in CG (p=0.038). There were no significant differences in relapse rates during pregnancy<br>Approximately twice as many smokers in the IG (43%) reported quitting smoking at 9 months, vs CG (25%) (p<0.01). At 6 weeks postpartum 25% of intervention baseline smokers were abstinent, vs 12% of control baseline smokers (p<0.01)<br>Participants "responded positively" to the programme. 88% of the IG completed at least 1 of the 12 behaviour change activity sheets. Staff were enthusiastic about the programme	1.1++I<br>1.2++I<br>1.8+I<br>2.3++I<br>3.1++I<br>5.1++IC<br>5.3++I<br>8.2++I<br>9.2++I<br>10.1+I<br>10.2+I<br>10.7++I<br>11.2++I<br>12.3++I
McBride 2004	Pregnant women (approached at ~11 weeks of pregnancy); 46% smokers and 54% recent quitters	3 arm trial:<br>IG 1: Partner-assisted (PA) intervention. Usual care plus a late-pregnancy relapse prevention kit (booklet and gift items) and	7-day self-reported abstinence at 28 weeks of pregnancy, 2, 6 and 12 months postpartum. Validated samples	No significant differences in women's abstinence at any follow-up. At 12 months postpartum, prevalence abstinence was 35% in IG 1; 32% in IG 2; and 29% in CG. In late pregnancy, more partners were abstinent in the PA condition (15%) vs usual care condition (5%) (p=0.02)	1.2++I<br>3.1++I<br>3.2++I (arm1)<br>3.3++I (arm1)

	(had smoked in the 30 days before pregnancy but were quit by baseline); plus partners for IG 1<br>Randomised: 193 IG 1; 192 IG 2; 198 CG<br>Analysed: 147 IG 1; 161 IG 2; 154 CG<br>USA	6 counselling calls (3 in each pregnancy trimester and 3 postpartum, at monthly intervals) delivered by a health advisor. Partners received telephone counselling and a support guide. Partners who smoked also received cessation aids and related counselling<br><br>IG 2: Women-only (WO). Usual care plus PA intervention minus partner support<br><br>CG: Usual care received provider advice to quit and a self-help guide	were obtained from women and partners not smoking in the previous 7 days at 28 weeks of pregnancy and 12 months postpartum. Also Partner Interaction Questionnaire to assess partner support	No significant differences in partner support by arm. In all arms women reported a decline in positive partner support from baseline to 12 months postpartum<br>Counsellors were trained; received a standard protocol and ongoing supervision<br>The number of calls received was significantly and positively associated with abstinence at 6 months postpartum (p=0.003). The intervention arm did not predict abstinence after controlling for number of calls, despite women in the PA condition receiving fewer calls than women in the WO condition (4 and 5 respectively, p=0.03)	5.3++IC<br>9.1++I<br>10.4+I
Morasco 2006	Pregnant women (<30 weeks gestation); 77% smokers or 23% recent quitters (quit during pregnancy or up to 30 days prior to learning they were pregnant); majority low income, Hispanic<br>Randomised: For	IG: Usual care plus psychotherapy relapse prevention treatment consisting of one 90-minute psychotherapy session, followed by bimonthly prenatal telephone calls during pregnancy, and monthly calls after delivery. Sessions were delivered by masters-level mental health counsellor clinicians trained in smoking cessation	Self-reported, biochemically validated 7-day point prevalence abstinence at end of pregnancy and 6 months postpartum. Also The Cigarette Timeline Follow-back Form (TLFB) to assess number of smoking days and cigarettes smoked per day (in	In the subgroup of baseline smokers: abstinence was 28.3% in IG and 9.6% in CG at the end of pregnancy (p=0.15). At 6 months postpartum abstinence was 9.4% in IG and 3.8% in CG (NS)<br>In the subgroup of spontaneous baseline quitters: the intervention had no effect on long-term abstinence. At pregnancy end, abstinence was 71% in IG and 84% in CG (NS). At 6 months postpartum abstinence was 43% in IG and 32% in CG (NS)<br>Counsellor training consisted of 16 hours initial group training and 24 hours of group clinical supervision focused on review of clinical cases<br>For the 53 baseline smokers randomised to the	1.2++I<br>3.1++I<br>4.2++I<br>5.1++IC<br>5.3++IC<br>9.1++IC

	current smokers: 53 IG; 52 CG. For spontaneous quitters: 14 IG; 19 CG<br>Analysed: Unclear; 19 current smokers lost to follow-up; 7 spontaneous quitter participants lost to follow-up<br>USA	CG: Usual care including an educational smoking cessation booklet for pregnant women; and health care provider quit smoking messages and reinforcement for smoking abstinence	past 30 days); one item from the Fagerstrom Test of Nicotine Dependence; Smoking Self-Efficacy Questionnaire	intervention, 36 received face-to-face counselling and 17 did not. The call schedule "proved to be unfeasible in this population". A mean of 2.6 telephone calls per patient were completed.<br>For baseline smokers; cost of the intervention was $56 per patient and cost to produce a non-smoker at pregnancy end was $299	
NEWS study (Newborns Excel Without Second hand Smoke) Winickoff 2010	Both parents of newborns (34% fathers) approached at delivery; 71% smokers and 29% recent quitters (smoked since 1 month before pregnancy)<br>Randomised: 48 IG; 53 CG<br>Analysed: 33 IG; 40 CG<br>USA	IG: 1) tailored in-hospital counselling during delivery hospital stay- one 15 minute in-person session delivered by trained study staff; 2) enrolment in a state proactive telephone counselling intervention; 3) letters to paediatrician, primary care provider and obstetrician recommending strategies to support parental cessation and ongoing support and NRT where appropriate; plus the control intervention<br><br>CG: Educational pamphlet about smoking and	Satisfaction with intervention components. Also self-reported, biochemically validated 7-day abstinence at 3 months postpartum	At 3 months postpartum, based on intention-to-treat analysis, biochemically validated abstinence was 9% in IG and 3% in CG (NS)<br>All parents in the IG received the in-hospital counselling session, 94% had a fax sent to a provider, and 36 (75%) accepted quitline enrolment. No one in the CG had direct contact with the quitline despite receiving the quitline contact information	3.1++I<br>5.3++IC<br>9.1+I

		secondhand smoke; and given contact information for the state quitline			
* PANDA (Parents and Newborns Developing and Adjusting) Mullen 2001	Abstinent pregnant women at 28th week of pregnancy (reporting not smoking in past 28 days); plus partners<br>Randomised: Unclear; 485-552 overall; 256 in CG<br>Analysed: Unclear; 5% of the CG could not be reached<br>USA	IG: Mailed newsletters and videos- women were sent 1 videotape and 5 newsletters; and partners were sent a different videotape and set of newsletters (sent from 28 weeks gestation to 6 weeks postpartum). Prior to the study participants received brief counselling and were mailed 8 self-help booklets weekly<br><br>CG: Standard care. Prior to the study participants received brief counselling and were mailed 8 self-help booklets weekly	Self-reported smoking status at 6 weeks, 3, 6 and 12 months postpartum (biochemically validated in a sample at 12 months postpartum). Also smoking attitudinal measures and exposure to environmental smoke	At 12 months postpartum, abstinence was significantly greater in IG than CG 55% vs 45%<br>Interviewers conducting smoking status telephone interviews were trained and 7% were evaluated, yielding "satisfactory" compliance with the protocol. Postcards to 15% of interviewees verified all (with a 67% return rate) had been interviewed<br>Evaluation explored in the pilot project reported 96% of women receiving videos and newsletters, 78% reading all newsletters and 66% watching videos	1.2++I<br>3.1++IC<br>4.1++I<br>5.1++I<br>5.3++IC<br>6.2++I<br>7.1++I<br>9.2+I<br>11.2++I<br>12.5+I<br>13.1++I<br>13.2+I
Peterson 1992	Pregnant women enrolling into prenatal care; smokers and recent quitters (quit within the previous 3 months)<br>Randomised: Unclear<br>Analysed: 71 IG;	IG: Usual care plus a pregnancy specific self-help manual and audiocassette and other pregnancy health education materials<br><br>CG: Usual care and mailed a list of community-based smoking cessation resources and other pregnancy health education	Self-reported smoking status at 6 months gestation and 8 weeks postpartum. Half of subjects reporting non-smoking at 6 months gestation were randomly selected to have a urine cotinine test	Significant differences only for smokers at baseline at 8 weeks postpartum; proportion of non-smokers was 29% in IG and 9.7% in CG (p=0.05)<br>Materials were mailed to patients by study personnel, no effort was made to modify clinicians usual practice<br>Cost of $50-111 per patient	1.2+I<br>5.3++IC<br>11.2++I

	78 CG<br>USA	materials			
Polanska 2004	Pregnant women (<36 weeks gestation); 77% smokers and 23% recent quitters (quit no later than 1 month before the visit)<br>Randomised: 216 IG; 194 CG<br>Analysed: 205 IG; 181 CG<br>Poland	IG: Four midwife home visits during pregnancy and one postpartum plus written translated materials prepared by the Community Health Research Unit in Ottawa<br><br>CG: Standard written information about the health risks of smoking to the fetus and benefits of smoking abstinence	Smoking status at each midwife visit for the IG; and for the CG, at the 20th week of pregnancy and delivery. Also infant birthweight. Self-reported smoking relapse one year after delivery was assessed by interview in a sample of 175 women who quit smoking during pregnancy	For baseline smokers, proportion of women who quit smoking was 44.3% in IG and 16.7% in CG ($p<0.001$)<br>All baseline quitters reported smoking abstinence at pregnancy end<br>One year postpartum there were no differences in smoking relapse between women who quit smoking due to participating in the intervention and control groups. For baseline quitters, risk of relapse one year postpartum was 3-fold lower in the IG than in CG. Quitters had significantly higher infant birthweights than smokers	1.2++I<br>5.1++C<br>5.3++C
Quit for Keeps Stretcher 2000	Pregnant women (approached at the 1st prenatal visit); smokers and recent quitters (quit since becoming pregnant)<br>Randomised: 88 IG; 85 CG<br>Analysed: Unclear<br>USA	IG: Tailored message intervention. Computer questionnaires determined tailored messages which were mailed after each prenatal visit<br><br>CG: Untailored self help guide	Self-reported biochemically validated smoking status at the 24th week of pregnancy; at 6 weeks postpartum and 3 months postpartum	No significant differences between IG and CG. Postpartum, in self-reported cessation using an intent-to-treat model, 9.6% in IG quit smoking vs 9.2% in CG<br>Of the women in the IG, 55% considered the information useful; 60% felt their needs were addressed; and 70% believed their barriers were addressed in the information	5.3++IC
Quit-for-Two Pollak 2016	Abstinent pregnant women (abstinent for at least 1 month	IG: One relapse prevention booklet plus one of two levels of stepped-care depending on assessment	Self-reported biochemically validated 7-day point prevalence	At 12 months postpartum, abstinence (intent-to-treat analysis) was 35% in IG and 36% in CG (NS)<br>For women at low risk of returning to smoking, crude abstinence rate was significantly higher in CG	1.1++I<br>1.2++IC<br>1.7++I<br>2.2+I

	prior to baseline)<br>Randomised: 188 IG; 194 CG<br>Analysed: Unclear<br>USA	of bio-behavioural risk of returning to smoking (based on nicotine dependence, intention to return to smoking and confidence to remain quit). Those at lower risk were offered 1 in-person session and 1 phone call during the 3rd trimester of pregnancy and 7 calls postpartum over 9 months (spaced "more tightly" in earlier postpartum). Those at higher risk were offered 1 in-person session and 2 calls during the third trimester and 11 calls postpartum over 9 months. Sessions were delivered by nurse counsellors<br><br>CG: One relapse prevention booklet plus 11 monthly newsletters with general health messages for pregnant and postpartum women (containing no smoking specific information)	abstinence. Also continuous abstinence at 6 weeks, 6 months and 12 months postpartum. Daily smoking was assessed using Time Line Follow-back Recall	(46%) than in IG (33%). For women at high risk of returning to smoking, crude abstinence rate was slightly lower but not different in CG (31%) vs IG (37%)<br>Counsellors were trained and had to complete a certification counselling session with a supervisor before providing counselling. Sessions were audio-recorded and supervisors listened to ~10% of cases. Feedback was provided based on Motivational Interviewing Treatment Integrity Scale. Counsellors met the supervisor bi-weekly, and the supervisor was available for consultations between times<br>Counsellors rated participant engagement (1= not engaged to 7= completely engaged). Mean engagement was 6.3 ± 0.4	3.1++I<br>4.1++I<br>5.3++IC<br>9.1++I<br>11.2+I<br>15.1++I
QT (Quit Together) Pbert 2004	Pregnant women (<2 months before due date); 71% smokers and	IG: Brief tailored stage of change counselling and materials at baseline, end of pregnancy, 3 and 6 months	Self-reported, biochemically validated 7-day abstinence before	Significant differences only stratifying by baseline smoking status. At pregnancy end, for smokers at baseline, 30-day abstinence was 26% in IG and 12% in CG (p=0.05); and at 1 month postpartum 26% vs	1.2++I<br>1.9+I<br>4.1+I<br>5.3++I

	29% recent quitters (quit after learning of pregnancy)<br>Randomised: 309 IG; 300 CG<br>Analysed: 120 IG; 161 CG<br>USA	postpartum. Sessions delivered by obstetric, paediatric, and Women, Infants and Children (WIC) providers<br><br>CG: Usual care	delivery; 1, 3 and 6 months postpartum. Also tobacco use and variables affecting smoking status. At the end of pregnancy women were considered smokers if self-reporting smoking in the 30 days prior to delivery	11% (p=0.04). No significant differences at 3 and 6 months postpartum<br>Initial training and support materials given to intervention providers, and individual coaching and feedback given 2 months after initial training. The intervention co-ordinator attended staff meetings to reinforce intervention delivery<br>Women in CG were more likely to complete one of the pre-natal interviews (91% vs 79%); and postpartum interviews (81% vs 73%)<br>Low level of intervention implementation with falling rates over time e.g. asking about smoking dropped from 71% to 23% postpartum, and advising to quit/remain abstinent dropped from 39% to 4%. Surveys at clinic sites revealed a low awareness of smoking risks and NRT amongst staff, with lower knowledge correlated to lower performance. Clinic type, smoking-related knowledge, older age and perception of smoking cessation as a priority were all independently related to better counselling performance<br>Process evaluation found barriers to be: difficulty in identifying key individuals to ensure intervention implementation; poor or inconsistent communication and leadership; staff turnover and redundancy; pace of other work and view of priority of smoking; viewing the child as the patient rather than the parents; staff misunderstanding of the intervention and their role; and variability in record documentation	9.1++I<br>15.1+I
* Reitzel 2010	Abstinent pregnant women (in 30th-33rd	3 arm trial:<br>IG 1: MAPS Motivation and Problem Solving	Self-reported biochemically validated continuous	MAPS and MAPS+ groups were combined for analysis (since groups did not significantly differ on characteristics, counselling received or abstinence).	1.1+I<br>1.2++IC<br>3.1++IC

	week of pregnancy); spontaneously quit (within 2 months prior to pregnancy or during pregnancy before the 30th week of gestation); predominantly low income<br>Randomised: 68 IG 1; 68 IG 2: 115 CG<br>Analysed: 46 IG 1; 52 IG 2; 88 CG<br>USA	intervention- 6 telephone counselling sessions averaging 22 minutes in length, delivered by trained counsellors (weeks 24 and 36 prepartum and weeks 2, 4, 7 and 16 postpartum); plus usual care as for the control<br><br>IG 2: MAPS+; MAPS as for intervention 1 plus 2 additional in-person counselling sessions (baseline and week 8 postpartum); plus usual care as for the control<br><br>CG: Usual care including self-help materials and 5-10 minutes of relapse prevention advice provided by trained researchers	abstinence at weeks 8 and 26 postpartum. Also smoking rate, smoking environment, craving, depression and anxiety, self-efficacy, social support, prepartum motivation	At 8 weeks postpartum, abstinence was 41.9% in MAPS and MAPS + (Arms 1 and 2 combined) and 27.8% in CG. At 26 weeks postpartum, abstinence was 22.8% in MAPS and MAPS+ (combined) and 16.5% in CG. Difference was significant (p=0.05) in adjusted analysis (adjusted for age, ethnicity, partner status, education, smoking rate and smokers in environment) and was more efficacious among women with higher prequit smoking rates<br>Counsellors were trained in motivational interviewing and tobacco treatment specialist training (>40 hours). MAPS/MAPS+ protocol training was approximately 80 hours of manual review and role playing with a supervisor. Counsellors met twice monthly with supervisors for case review and practice, and supervisors were available for consultation between appointments. Counsellors sought feedback from participants throughout. Counselling sessions were recorded, and two randomly selected tapes/month/ counsellor were coded with an adapted version of the Motivational Interviewing Treatment Integrity (MITI) Code. Counsellors maintained "adequate" adherence to MI (empathy/understanding M = 5.39 [SD = 0.62] and MI spirit M = 5.50 [SD = 0.57]). Counsellors maintained "excellent" adherence to the MAPS/MAPS+ treatment manual (M = 6.77 [SD = 0.50]). 80% of MAPS/MAPS+ participants completed at least four counselling calls, 67% completed at least five, and 47% completed all six calls. Of those in MAPS+, 100% completed the baseline in-person counselling session and 81% completed the 8-week session	5.1++IC<br>5.3+IC<br>11.2+I

Ruger 2008	Pregnant women (<28 weeks); 81% smokers and 19% recent quitters (within 3 months of baseline); low income<br>Randomised: 156 IG; 146 CG<br>Analysed: 131 IG; 128 CG<br>USA	IG: Tailored motivational interviewing via 3 home visits (1 hour each on average, delivered monthly until 1 month postpartum) and self-help smoking manuals. Sessions delivered by outreach health nurses<br><br>CG: Usual care. Up to 5 minute intervention outlining the harmful effects of smoking during and after pregnancy and self-help manuals	Self-reported biochemically validated 30 day abstinence at 1 month after the intervention and 6 months postpartum. Also cost effectiveness and maternal and infant outcomes	At 6 months postpartum, for baseline quitters, abstinence was 43% in IG and 18% in CG (p=0.055). For baseline smokers, cessation rates were similar; 7/110 non-smokers at 6 months postpartum for IG and 8/100 for CG. No significant differences in infant health outcomes<br><br>The cost-effectiveness of motivational interviewing for relapse prevention compared to usual care was estimated at $851/QALY saved and $628/QALY saved. Including savings in maternal medical costs in sensitivity analysis resulted in cost savings for motivational interviewing for relapse prevention. For smoking cessation, motivational interviewing cost more but did not provide additional benefit vs usual care. Total cost of $311.8 per participant for the intervention vs $4.82 per participant for usual care. Main costs were intervention delivery, travel time, scheduling and training	1.1++I<br>1.5+I<br>4.1++IC<br>5.3++IC<br>2.6+I<br>9.1++IC<br>9.2++I<br>12.2+I<br>15.1++I
SCIP (Smoking Cessation in Pregnancy) Kendrick 1995	Pregnant women (approached at first prenatal visit); 85% current smokers or 15% recent quitters (not smoking at enrolment but who smoked within 7 days before awareness of pregnancy)<br>Randomised: Unclear; 5572	IG: Printed materials and brief counselling<br><br>CG: "usual care" delivered by clinics	Self-reported, biochemically validated smoking status in the eighth month of pregnancy and 6-12 weeks postpartum. Also birthweight<br>Opinion forms were collected from a subset of women to assess exposure to the intervention and comments from	For recent quitters at enrolment, the "majority" did not resume smoking by the eighth month, although verified rates of abstinence were lower than self-reported rates (data not reported)<br>For enrolment smokers, at the eight month of pregnancy, cotinine-verified quit rates were not significantly different between intervention and control sites (6.1% vs 5.9%), although more enrolment smokers attending intervention clinics were likely to report quitting smoking. Post-hoc analysis showed a statistically significant effect only among women who stated at enrolment they did not think they would quit smoking (p=0.023)<br>The intervention had no effect on birthweight	1.2++I<br>2.6+I<br>3.1++I<br>5.3++I<br>9.1+I

	overall (1741 Colorado, 1936 Maryland, 1895 Missouri)<br>Analysed: Unclear; 45% loss to follow up<br>USA		providers were sought	The majority of intervention women reported receiving study materials and counselling, but also reported receiving non-study smoking cessation materials and counselling<br>Clinic staff were enthusiastic about the intervention but found the data collection time-consuming	
Secker-Walker 1995	Abstinent pregnant women (quit by first prenatal visit). Part of a wider cessation trial- current smokers were assigned to a separate smoking cessation intervention<br>Randomised: 89 IG; 86 CG<br>Analysed: For long-term postpartum cessation rates; 55 IG; 52 CG<br>USA	IG: Smoking relapse prevention counselling from a trained counsellor at the 1st, 2nd and 3rd prenatal visits, 36-week prenatal visits and 6 week postpartum visits (10-20 minutes per session); plus usual care<br><br>CG: Usual care about smoking or staying abstinent provided from obstetricians or midwives	Self-reported smoking status at 36 weeks gestation (biochemically verified at 36 weeks); and self-reported status at long-term postpartum follow-up, up to 54 months postpartum (for the IG also recorded at the 2nd and 3rd prenatal visits). Also predictors of relapse; birthweight and complications	At 36 weeks, 8.8% in IG reported smoking versus 16.9% in CG (NS).No significant differences based on biochemically validated status. At long-term, postpartum relapse rates were not significantly different: 50.9% in IG and 50% in CG. The average number of days abstinence reported by women in IG was significantly longer than in CG (199 days vs 166 days p<0.01)<br>No significant differences in birthweight or complications<br>All but 3 of the women not lost to follow-up in the IG participated in the 3 individual prenatal counselling sessions, and 93% were counselled postpartum	1.2++I<br>3.1++I<br>4.1++I<br>5.1++I<br>9.1++IC<br>9.2++I<br>13.1++I
Secker-Walker 1998	Abstinent pregnant women (quit by their first prenatal visit); predominantly	IG: Structured physician advice plus referral to an on-site relapse prevention counsellor provided by a trained nurse (at the 1st,	Self-reported, biochemically validated smoking status during pregnancy; and self-	At 36 weeks, relapse rates were 23% in both groups. At 1 year postpartum, relapse was 32% in IG and 22% in CG (NS). In intention-to-treat analysis, relapse rate was 55% in IG and 48% in CG<br>Among women in IG not lost to follow-up, all	1.2++I<br>3.1++IC<br>5.3++IC<br>9.1++IC<br>10.4+IC

	low income. Part of a wider cessation trial- current smokers were assigned to a separate smoking cessation intervention<br>Randomised: 62 IG; 63 CG<br>Analysed: 37 IG; 41 CG<br>USA	2nd, 3rd and 5th prenatal visits and at the 36 week visit)<br><br>CG: Usual physician advice plus a self-help booklet	report 1 year postpartum. Also smoking attitudinal measures	received counselling at the 1st visit, 95% at the 2nd visit, 86% at the 3rd visit, 66% at the 5th visit, and 100% at the 36-week visit. Almost all women in each group reported that their physicians had talked about smoking at the 1st visit. At the 2nd and 36-week visits, significantly smaller proportions of women in CG reported that their physician had talked about smoking. For IG, perceptions of concern shown by their physician about smoking were maintained at a high level. For CG, these perceptions were significantly lower, and fell during pregnancy	
STARTS (Strategies to Avoid Returning to Smoking) Levine 2016	Abstinent pregnant women (approached in the third trimester); recent quitters (had not smoked in the past two weeks); predominantly low-income<br>Randomised: 150 IG; 150 CG<br>Analysed: 127 IG; 139 CG<br>USA	IG: STARTS intervention (Strategies to Avoid Returning to Smoking). Individualised enhanced cognitive behavioural intervention (13 sessions), including written materials, focused on women's postpartum concerns about mood, stress and weight. Sessions initiated at delivery until 24 weeks postpartum and delivered by clinicians, alternating between in person (mean 26 minutes duration) or telephone contact (mean 14 minutes duration)	Self-reported biochemically validated sustained abstinence at 52 weeks postpartum. Also self-reported mood (Center for Epidemiologic Studies Depression Scale), stress (Perceived Stress Scale), and self-efficacy for weight management and smoking specific weight concerns at 12, 24 and 52 weeks postpartum	Overall 38%, 34% and 24% of the sample maintained abstinence at 12, 24 and 52 weeks respectively. There was no differences between groups in abstinence or time to relapse. Self-reported depression (p<0.001) and perceived stress (p=0.001) significantly improved over time and improvements were similar for both groups. In both groups, there was a decrease in weight concern over time (p=0.02); with significantly lower concerns in STARTS vs SUPPORT at 24 weeks postpartum<br>Clinicians received treatment manual and received weekly group supervision. Sessions were audio-recorded and reviewed by independent raters. Overall 91% of sessions reviewed were rated as compliant with the protocol. Significantly more SUPPORT sessions were rated as compliant vs STARTS sessions (p=0.003). 86% of women completed all 13 intervention sessions. Across	1.1++I<br>1.2++IC<br>1.5++I<br>1.9+IC<br>2.3++IC<br>3.1++IC<br>4.1++I<br>4.2++I<br>5.1++IC<br>5.3++IC<br>6.2+I<br>8.1+I<br>8.2++I<br>9.1+IC<br>9.2++I<br>11.2++I<br>13.2++I<br>16.2+IC

		CG: SUPPORT intervention (supportive time and attention comparison). Individualised cognitive behavioural intervention (13 sessions), including written materials, as for STARTS, though focused only on behavioural urges to smoke. Sessions initiated at delivery until 24 weeks postpartum and delivered by clinicians, alternating between in person or telephone contact		interventions, women completed more in-person sessions than telephone sessions	
Suplee 2005	Abstinent pregnant women (in the third trimester); quitters (self-reported quitting during pregnancy or in the 3 months before); predominantly African American<br>Randomised: 30 IG; 32 CG<br>Analysed: Unclear; 53 overall<br>USA	IG: Brief counselling session (10-20 minutes) delivered by a researcher in the immediate postpartum period and educational materials; plus standard care<br><br>CG: Standard care (including no smoking cessation information)	Self-reported, biochemically validated relapse to smoking at 4-8 weeks postpartum. Also Relapse Prevention Questionnaire (SRPQ) to measure smoking exposure	At 4-8 weeks postpartum, based on intention-to-treat analysis, 37% in IG were cotinine negative and 25% in CG were cotinine negative (NS)<br>Significant difference between self-reported and biochemically validated smoking status	1.2++I<br>1.4++I<br>3.1++I<br>5.1++I<br>5.3++I<br>7.1++I<br>9.1++I<br>10.4++I<br>15.1++I

The Rotunda Stop Smoking Programme Thornton 1997	Pregnant women (at the first prenatal visit); 94.5% smokers and 5.5% recent quitters (quit prior to entering prenatal care); majority low SES status; plus partners<br>Randomised: 209 IG; 209 CG<br>Analysed: 167 IG; 174 CG<br>Ireland	IG: One to one counselling by a trained midwife stop smoking facilitator (10-15 minutes at the first session and available at subsequent prenatal sessions) plus booklets and CO monitor; plus support groups; and routine care as for the control group. Support offered to both women and partners<br><br>CG: Routine care and advice (including smoking related advice)	Feasibility and effectiveness. Also self-reported point prevalence abstinence at delivery and 3 months postpartum; and infant outcomes	At delivery, 12% in IG were abstinent and 13% in CG (NS). At 3 months postpartum, 11% in IG were abstinent and 7% in CG (NS). No significant differences in gestation or birthweight between groups<br>89% of IG reported the programme to be of "some" or "good" benefit; 57% recommended continuing the programme and 33% recommended continuing with some changes. Issues raised were the antenatal clinic not being ideal for intervention delivery due to the length, and lack of privacy. Some women recommended provision of more support. Key worker observations included, women rarely took up further counselling unless initiated by the key worker. Due to low attendance at stop smoking support groups this part of the intervention was later dropped	1.2++I<br>2.6++IC<br>3.1++I<br>5.1++I<br>5.3++I<br>9.1++IC<br>15.1++I

+=probably present; ++=definitely present; I=intervention; C=control; IC=intervention and control groups

* Trials which had long-term effectiveness

Abbreviations: IG= intervention group; CG=control group; NS= not significant
